# Assessing the post-treatment therapeutic effect of pinaverium in irritable bowel syndrome: a randomized controlled trial

**DOI:** 10.1038/s41598-021-92990-7

**Published:** 2021-07-06

**Authors:** Liang Zheng, Weimin Lu, Qi Xiao, Yaoliang Lai, Heng Fan, Yuling Sun, Dawei Huang, Yuanyuan Wang, Zhen Li, Zhengyan Jiang, Xingxing Liu, Lijuan Zhang, Dongmei Zuo, Zhexing Shou, Qing Tang, Huisuo Huang, Yongqiang Yang, Zongxiang Tang, Jun Xiao

**Affiliations:** 1 Department of Gastroenterology, The Second Affiliated Hospital of Nanjing University of Chinese Medicine, 23 Nanhu Rd, Nanjing, 210017 China; 2 Department of Internal Medicine, Jiangsu Provincial Hospital of Chinese Medicine, Affiliated Hospital of Nanjing University of Chinese Medicine, 155 Hanzhong Rd., Nanjing, 210029 China; 3 The School of Medicine, Washington University, 660 S Euclid Ave., St. Louis, MO 63110 USA; 4 Department of Gastroenterology, Beijing Xuanwu Hospital of Chinese Medicine, 8 Wanming Rd., Beijing, 100050 China; 5 Department of Integrated Chinese Medicine and Western Medicine, Union Hospital, Tongji Medical College, Huazhong University of Science and Technology, 1277 Liberty Rd., Wuhan, 430022 China; 6 The State Key Laboratory Cultivation Base for TCM Quality and Efficacy, The School of Medicine and Life Sciences, Nanjing University of Chinese Medicine, 138 Xianlin Road, Nanjing, 210023 China; 7 The Macrohard Institute of Health, 231 North Ave., Battle Creek, MI 49017 USA

**Keywords:** Gastroenterology, Medical research

## Abstract

Irritable bowel syndrome (IBS) is the most common gastrointestinal disorder significantly decreasing patients’ lives of quality and placing huge economic burden on our society. Existing studies indicated that the therapeutic effects maintained for a period of time after the treatments were discontinued. It is clinically important to assess these post-treatment therapeutic effects (PTTE), which prevent IBS from relapsing. To assess the PTTE in pinaverium treatment and obtain high-quality evidence to justify the use of PTTE for long-term IBS management, we performed this controlled, double blind study on patients with IBS who were randomized to pinaverium 50 mg (n = 132) or placebo (n = 132), three times daily, for 4 weeks, and were followed up for 57 weeks after the treatments. The primary endpoints were abdominal pain and stool consistency. The secondary endpoints were pain frequency and stool frequency. The tertiary endpoints were global overall symptom and adverse events. Three days after pinaverium was discontinued, endpoints rebounded only 23.2–42.8% (*P* < 0.015 cf. placebo). The PTTE (*P* < 0.05 cf. placebo) lasted 9–17 weeks, which is similar to other antispasmodics with a 15-week treatment in striking contrast to ≥ 1 year PTTE in cognitive behavior therapy and < 1 week PTTE in serotonin antagonist treatment indicating that PTTE length markedly depends on the medication class used for the treatment and less depends on treatment length. After 17 weeks, the stage could be considered as an IBS natural history [no significant differences between pinaverium and placebo (all endpoints’ *P*’s > 0.05)], during which an average of 51.5–56.4% of patients (pool pinaverium and placebo data together) had IBS symptoms. These results provide clinical insights into efficient and cost-effective management of refractory IBS, and lend support to the IBS management that the selection of a therapy should consider both its effectiveness during treatment and its PTTE after the treatment.

Trial registration number: NCT02330029 (16/08/2016).

## Introduction

Irritable bowel syndrome (IBS) is the most common chronic and a highly relapsing gastrointestinal disorder with an estimated worldwide prevalence of 10–15%^1^. More than 50% of patients were still symptomatic with IBS, and a further 25% of the patients had minor IBS symptoms after 1 and 7 years^2^. For many patients, IBS is a lifelong condition^3^. Therefore, how to prevent IBS from relapsing after the treatment is equally important to how effective the therapy is during the treatment.

Existing studies showed that the therapeutic effects maintained for a period of time after the treatments were discontinued, or IBS treatments have post-treatment therapeutic effects (PTTE) that prevent the recurrence of IBS^4^. However, only a few studies investigated the PTTE (Supplemental Material Table S1); very few studies followed up with patients after PTTE disappeared (Table S2); and no studies have been specifically designed to assess PTTE. In particular, of the 188 randomized controlled trials reviewed by the American College of Gastroenterology, only 31 trials collected outcome data after treatments and compared the post-treatment data with those of a placebo/control^5^. And although these studies collected post-treatment data, they were not intended to investigate the PTTE. Some studies’ post-treatment follow-up duration were too short to cover the entire PTTE (trail # 1, 3, 5–20, 23–25 in Table S2). Cappello et al. and Vahedi et al. showed that the PTTE was still significant (*P* < 0.05) at week 4 after treatment, but no data were collected after week 4 so that the accurate length of PTTE was still unknown^6,7^. The most accurate conclusion we could draw from this study was that the PTTE length was 4 weeks or longer. Some studies only collected post-treatment data 1 year later (trail # 21) or 6 months later (trail # 22) while no data were collected in between. This data collection resolution was too low to accurately identify the PTTE length. For example, the PTTE length of paroxetine in the study by Creed et al. could be anywhere from < 1 week to 11 months because Creed et al. only collected the post-treatment data 1 year after the therapy was discontinued^8^.

Nevertheless, the preliminary data from existing studies interestingly showed that the length of PTTE markedly depended on the types of treatment (“treatment-dependent PTTE”) (Table S2). Cognitive behavior therapy (CBT) had the longest PTTE (roughly one year or longer), while 5-hydroxytryptamine (5-HT_3_) antagonist, alosetron, the shortest (less than one week), and antidepressants had 4 weeks to 1 year PTTE^7–11^. Strikingly remarkable is that in CBT, the PTTE length less depends on the treatment length, who administered the treatment, and how the treatment was administered. Heitkemper et al. demonstrated that 8 weekly 1-h sessions self-management CBT (Comprehensive group) had a similar PTTE length to one 90-min session self-management CBT (Brief group) that covered the same material as the Comprehensive group^9^. Lackner et al. showed that 10 session therapist-administered CBT and 4 session self-administered CBT had similar lengths of PTTE^10^. Everitt et al. demonstrated that telephone-CBT and web-CBT had similar lengths of PTTE^11^. Jarrett et al. showed that CBT delivered in-person and CBT delivered via telephone had similar lengths of PTTE^12^. All these results provide clinically and socially important insight into cost-effective long-term management of refractory IBS.

PTTE is increasingly recognised as being valuable for decreasing the recurrence of IBS^13^. To accurately assess PTTE, a study should (1) test simultaneously whether the treatment is effective when compared with a placebo, (2) follow up with patients for a period that is long enough to cover both the PTTE period, if any, and the IBS natural history thereafter, (3) collect data at an adequate resolution. This study satisfies this 3-element criterion.

Antispasmodics are one of the common IBS medications^14^. Clavé et al. showed that patients on otilonium for 15 weeks benefited from a 10-week or more PTTE^15^. Pinaverium is one of the most commonly used IBS medications worldwide^16–22^. Our previous study demonstrated that pinaverium for 4 weeks effectively relieved IBS symptoms^16^. To further investigate the PTTE of this pharmacological therapy, the relapse-free probability, and the IBS natural history after the PTTE, this study collected post-treatment data on days 1, 2, and 3, at weeks 4, 9, 17, 25, 33, 45, and 57.

## Methods

### Trial design and settings

This study was conducted at four hospitals in China from December 2016 to June 2019. The protocol was approved by the Institutional Review Board of each participating hospital and by the Ethics Committee of the Macrohard Institute of Health. The Institutional Review Board guidelines for clinical research were strictly followed. Research staff recruited potential participants, and explained the purpose and eligibility requirements of the study to them. Written consent was obtained from each subject prior to enrollment. A questionnaire regarding patients’ medical history was administered (Table S3). This study consisted of 2 weeks of run-in, 4 weeks of treatment, and 57 weeks of follow-up.

### Diagnosis, inclusion, and exclusion criteria

Subjects who were diagnosed with diarrhea-predominant IBS by the Rome IV criteria were eligible for enrollment. Specifically, patients had recurrent abdominal pain on average at least 1 day/week during the previous 3 months that is associated with 2 or more of the following: (a) related to defecation, (b) associated with a change in stool frequency, and/or (c) associated with a change in stool appearance. The criterion was fulfilled for the last 3 months with symptom onset at least 6 months prior to diagnosis. The inclusion criteria and exclusion criteria were described in our previous trial^16^.

### Randomization and blinding

The permuted block randomization sequence was computer generated with a block size of six. The sequence was concealed in an opaque envelope, and was kept by the independent assistants at each participating hospital until the treatment was assigned. All investigators were blinded to the randomization sequence. After obtaining written consents from eligible subjects, the study nurses contacted the independent assistants to obtain an envelope, which contained the allocation information. Eligible subjects were randomized to receive pinaverium, 50 mg tablets, or placebo, which was visually identical in appearance to pinaverium. Both were taken three times daily for 4 weeks.

### Study outcomes

The primary and secondary endpoints recommended by the US Food and Drug Administration for IBS were used in this study^23^. Briefly, the primary endpoints were the average of pain intensity (0 = no pain, 10 = worst pain) and stool consistency (Bristol stool form scale) in the worst day of the 30 days before treatment (baseline), or of the days between last follow-up and the current follow-up (treatment and post-treatment). The secondary endpoints (scale 0–10) included the averages of daily frequencies of abdominal pain and stool. A clinical responder was defined previously^16,23^.

The tertiary endpoints were adverse events and IBS global overall symptom scale^23^, which was evaluated based on the answer to the question “how would you overall rate your IBS symptoms today?” Zero indicates no symptoms; 1 indicates minimum symptoms; 2 indicates mild symptoms; 3 indicates moderate symptoms; 4 indicates severe symptoms.

The above clinical data were collected before treatment (baseline), during treatment (days 1, 2, and 3, weeks 1 and 4), and after treatment (days 1, 2, and 3, weeks 4, 9, 17, 25, 33, 45, and 57). A bowel symptom scale table containing the above endpoint questionnaires was developed for the trial (Table S4).

### Statistical analysis

Categorical data were compared using the Chi-square test. Continuous data were expressed as mean ± standard deviation and compared using the Student’s t test. The sample size was estimated using power calculation (see the Supplemental Materials). All *p* values were two-tailed with the level of statistical significance set at 0.05. The primary efficacy analysis included a comparison of the response rates between pinaverium and placebo. Pearson product moment correlation or the Average Inter-item Correlation (r_∑_) analyses were used to test the correlation among the primary endpoints, the secondary endpoints, and the global overall symptom scale. Logrank Tests (Kaplan–Meier Curves) were used for survival (relapse-free) analysis. All authors had access to the study data, and reviewed and approved the final manuscript.

## Results

### Patient populations and baseline characteristics

**Figure 1:**
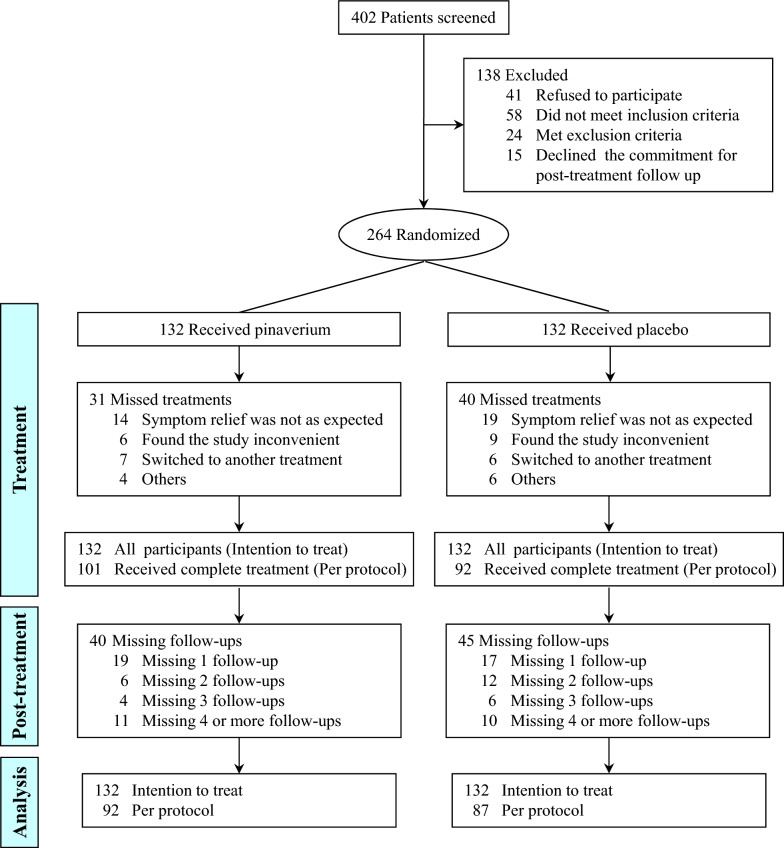
Flowchart of the trial.

A total of 402 patients were screened for this study (Fig. 1). Two hundred sixty-four patients were randomized to either take pinaverium or placebo. The intent-to-treat population consisted of 132 patients taking pinaverium and placebo, respectively, which were analyzed further. Baseline characteristics of the two groups showed no significant difference (*P* > 0.05) (Table 1).

**Table 1:** The baselines of demographics, IBS medical history, and IBS symptoms (intention-to-treat population).

Characteristic	Pinaverium (n = 132)	Placebo (n = 132)	*P* value
Age, mean ± SD	41.8 ± 14.0	38.9 ± 13.7	0.09
Male, n (%)	54 (41%)	51 (39%)	0.71
Female, n (%)	78 (59%)	81 (61%)	
IBS history, years ± SD	4.92 ± 4.42	4.98 ± 3.91	0.91
Abdominal pain, mean ± SD	4.92 ± 1.50	5.03 ± 1.62	0.58
Stool consistency, mean ± SD	6.10 ± 0.83	6.01 ± 0.76	0.35
Pain frequency, mean ± SD	4.66 ± 1.54	4.56 ± 1.54	0.60
Stool frequency, mean ± SD	4.36 ± 1.06	4.15 ± 1.09	0.12
Global overall symptom scale, mean ± SD	2.55 ± 0.89	2.55 ± 0.81	0.94

Chi-square for genders; T-test for others.

### Primary and secondary outcomes during the treatment

#### The onset of action

**Figure 2:**
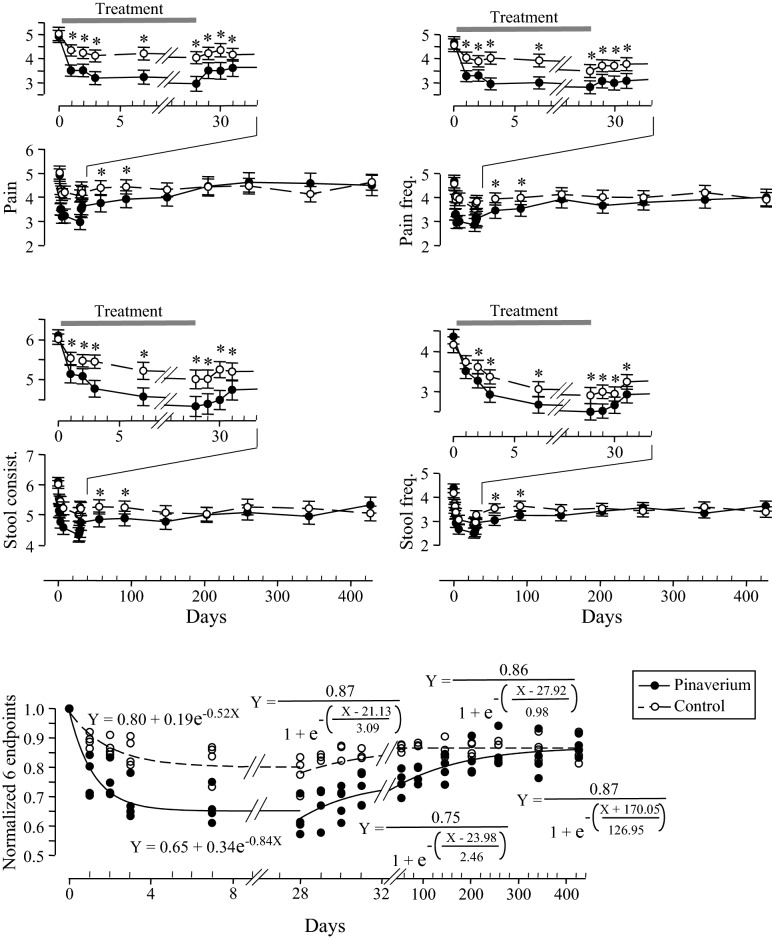
The time course of the endpoints during the treatment and post-treatment (intention-to-treat population; n = 132 for each group). Error bars indicate 95% confidence intervals. The numeric values of the endpoints were listed in Supplemental Material Tables S5 (intention-to-treat population) and S6 (per-protocol population). The *t* test comparing pinaverium with placebo is indicated by * (*P* < 0.05). Bottom panel: Responses of each endpoint during the treatment and post-treatment were normalized.

Pinaverium significantly and rapidly reduced the scales of the primary and secondary endpoints from day 1 (*P* < 0.05; Fig. 2; Tables S5 and S6). The decreases in the scales during the first 3 days accounted for 88.7% (pain intensity), 75.5% (stool consistency), 94.5% (pain frequency), and 77.6% (stool frequency) of the total decreases, respectively, [(Baseline − Day 3)/(Baseline − Week 4)] indicating that pinaverium relieved pain more quickly than improved stool consistency. No symptoms were significantly improved further after 1 week (*P* = 0.15–0.85) although the symptom scales still decreased after 1 week in the pinaverium group. These results indicate that pinaverium has a rapid onset of action for relieving IBS symptoms.

#### The efficacy of the treatment

Significantly more patients in the pinaverium group were clinical responders to the primary and secondary endpoints when compared with placebo (Table 2, *P* < 0.05–0.001). In particular, pinaverium relieved pain in 52.3%, 54.5%, 60.6%, 53.8%, and 64.4% of patients on days 1, 2, and 3, at weeks 1 and 4 while placebo in 17.4%, 23.5%, 29.5%, 30.3%, and 31.8% of the patients; pinaverium improved stool consistency in 28.8%, 31.8%, 37.9%, 47.7%, and 54.5% of patients on days 1, 2, and 3, at weeks 1 and 4 while placebo in 12.9%, 14.4%, 15.9%, 28.8%, and 31.8% of the patients.

**Table 2:** Efficacy of the treatment measured by endpoint symptoms (the response rates, odds ratios (OR), and 95% confidence interval (CI)) in the intention-to-treat population).

	Primary endpoints				Secondary endpoints			
	Pain		Stool consistency		Pain frequency		Stool frequency	
	Res%	OR (95% Cl) *P* (*X*_*2*_^2^)	Res%	OR (95% Cl) *P* (*X*_*2*_^2^)	Res%	OR (95% Cl) *P* (*X*_*2*_^2^)	Res%	OR (95% Cl) *P* (*X*_*2*_^2^)
**TREATMENT**								
Day 1								
Pinaverium	52.3	5.19	28.8	2.73	44.7	3.46	28.0	1.97
Placebo	17.4	(2.5–9.13)	12.9	(1.45–5.15)	18.9	(1.99–6.02)	16.5	(1.08–3.57)
		< 0.001 (35.3)		< 0.005 (10.1)		< 0.001 (20.2)		< 0.05 (5.0)
Day 2								
Pinaverium	54.5	3.91	31.8	2.68	50.8	2.75	42.4	2.86
Placebo	23.5	(2.30–6.63)	14.4	(1.46–4.93)	27.3	(1.65–4.59)	20.5	(1.66–4.94)
		< 0.001 (26.8)		< 0.005 (10.4)		< 0.001 (15.3)		< 0.001 (14.8)
Day 3								
Pinaverium	60.6	3.67	37.9	3.22	56.8	3.95	42.4	2.10
Placebo	29.5	(2.20–6.12)	15.9	(1.80–5.78)	25.0	(2.34–6.66)	26.0	(1.25–3.53)
		< 0.001 (25.7)		< 0.001 (16.2)		< 0.001 (27.6)		< 0.005 (7.9)
Week 1								
Pinaverium	53.8	2.68	47.7	2.26	60.6	2.88	47.0	2.07
Placebo	30.3	(1.62–4.44)	28.8	(1.36–3.76)	34.8	(1.74–4.74)	29.9	(1.25–3.44)
		< 0.001 (14.9)		< 0.005 (10.0)		< 0.001 (17.6)		< 0.005 (8.1)
Week 4								
Pinaverium	64.4	3.88	54.5	2.57	66.7	3.87	54.5	1.91
Placebo	31.8	(2.32–6.46)	31.8	(1.56–4.25)	34.1	(2.32–6.44)	38.6	(1.17–3.12)
		< 0.001 (28.1)		< 0.001 (13.9)		< 0.001 (28.0)		< 0.010 (6.8)
**POST-TREATMENT**								
Day 1								
Pinaverium	62.3	4.04	58.3	3.11	65.2	4.15	58.3	2.09
Placebo	29.5	(2.42–6.76)	31.1	(1.87–5.15)	31.1	(2.48–6.94)	40.2	(1.28–3.41)
		< 0.001 (29.5)		< 0.001 (19.9)		< 0.001 (30.7)		< 0.005 (8.7)
Day 2								
Pinaverium	62.1	4.37	56.8	3.25	63.6	4.33	59.1	2.22
Placebo	27.3	(2.60–7.36)	28.8	(1.95–5.42)	28.8	(2.58–7.26)	39.4	(1.36–3.64)
		< 0.001 (32.4)		< 0.001 (21.1)		< 0.001 (32.2)		< 0.005 (10.3)
Day 3								
Pinaverium	62.1	4.21	54.5	2.66	63.6	4.03	55.3	2.11
Placebo	28.0	(2.51–7.07)	31.1	(1.61–4.41)	30.3	(2.41–6.73)	37.0	(1.29–3.45)
		< 0.001 (31.0)		< 0.001 (14.9)		< 0.001 (29.4)		< 0.005 (8.9)
Week 4								
Pinaverium	56.8	2.63	47.0	1.71	59.8	3.31	53.0	2.21
Placebo	33.3	(1.60–4.34)	34.1	(1.04–2.81)	31.1	(1.99–5.49)	33.9	(1.34–3.62)
		< 0.001 (14.7)		< 0.05 (4.5)		< 0.001 (22.1)		< 0.005 (9.9)
Week 9								
Pinaverium	50.8	1.69	50.0	1.69	49.2	1.64	49.2	1.77
Placebo	37.9	(1.04–2.76)	37.1	(1.04–2.77)	37.1	(1.01–2.69)	35.4	(1.08–2.90)
		< 0.05 (4.5)		< 0.05 (4.5)		< 0.05 (4.0)		< 0.05 (5.2)
Week 17								
Pinaverium	43.9	1.47	47.0	1.55	47.0	1.55	45.5	1.37
Placebo	34.8	(0.89–2.41)	36.4	(0.95–2.54)	36.4	(0.95–2.54)	37.8	(0.84–2.24)
		> 0.05 (2.3)		> 0.05 (3.1)		> 0.05 (3.1)		> 0.05 (1.6)
Week 25								
Pinaverium	45.5	1.56	43.9	1.47	46.2	1.55	41.7	1.30
Placebo	34.8	(0.95–2.56)	34.8	(0.89–2.41)	35.6	(0.95–2.55)	35.4	(0.79–2.14)
		> 0.05 (3.1)		> 0.05 (2.3)		> 0.05 (3.1)		> 0.05 (1.1)
Week 33								
Pinaverium	40.2	1.34	41.7	1.59	43.2	1.57	42.4	1.44
Placebo	33.3	(0.81–2.22)	31.1	(0.96–2.63)	32.6	(0.95–2.60)	33.9	(0.87–2.37)
		> 0.05 (1.3)		> 0.05 (3.2)		> 0.05 (3.2)		> 0.05 (2.1)
Week 45								
Pinaverium	38.6	1.07	43.9	1.57	40.9	1.38	40.9	1.31
Placebo	37.1	(0.65–1.75)	33.3	(0.95–2.58)	33.3	(0.84–2.29)	34.6	(0.79–2.15)
		> 0.05 (0.1)		> 0.05 (3.1)		> 0.05 (1.6)		> 0.05 (1.1)
Week 57								
Pinaverium	37.1	1.10	40.9	1.29	39.4	1.30	42.4	1.30
Placebo	34.8	(0.67–1.82)	34.8	(0.76–2.13)	33.3	(0.79–2.15)	36.2	(0.79–2.13)
		> 0.05 (0.1)		> 0.05 (1.0)		> 0.05 (1.0)		> 0.05 (1.1)

*Res%* response rates.

The relative risks (RRs) for pain scale > 3 in patients of the pinaverium group were 0.74 (95% CI, 0.66–0.83), 0.74 (0.65–0.84), 0.70 (0.61–0.81), 0.70 (0.60–0.81), and 0.61 (0.51–0.72) when compared with placebo on days 1, 2, 3, and at weeks 1, 4 (Table S7), respectively, indicating that patients would be 1.6 times more likely to suffer moderate or severe pain at the end of the treatment if they had not taken pinaverium. The RRs for diarrhea (Bristol scale > 4) was 0.82 (0.72–0.93), 0.81 (0.70–0.92), 0.74 (0.63–0.86), 0.73 (0.60–0.89), and 0.67 (0.53–0.83) when compared with placebo during the treatment indicating that patients would be 1.5 times more like to suffer diarrhea if they had not taken pinaverium.

#### The correlation among the primary and secondary endpoints

The Average Inter-item Correlation (r_∑_) analysis indicated that pinaverium tended to relieve all the four symptoms simultaneously (Table S8). The correlations among the primary and secondary endpoints were gradually increased from moderate on day 1 to strong at week 4 (r_∑_ = 0.520–0.914), indicating that pinaverium relieved the four IBS symptoms simultaneously, but some symptoms were improved faster than others. The strongest correlation existed between one symptom and its frequency while weak correlation existed between different symptoms (Table S8).

### Primary and Secondary outcomes during the post-treatment

#### The offset of action

Pinaverium had a slow offset of action (Fig. 2). Three days after pinaverium was discontinued, the average pain scale rebounded only 42.8% while stool consistencies rebounded 41.2% [(Day 31–Day 28)/(Day 427–Day 28)]. These 3-day rebounds in the secondary endpoints were 23.2% (pain frequency) and 38.3% (stool frequency).

#### The maintenance of the efficacy after pinaverium was discontinued

The therapeutic effects of pinaverium were maintained until 9–17 weeks after pinaverium was discontinued. During this PTTE period, the symptomatic endpoints of the pinaverium group were significantly deceased when compared with those in the placebo group (*P* < 0.05; Fig. 2, Tables S5 and S6), the response rates in the pinaverium group were significantly higher than those in the placebo group (*P* < 0.05; Table 2), and the risk that patients suffered moderate or severe symptoms and the percentages of patients with IBS symptoms in the pinaverium group were significantly lower than those in the placebo group (*P* < 0.05; Tables S7 and S9).

### Survival (relapse-free) analysis

**Figure 3:**
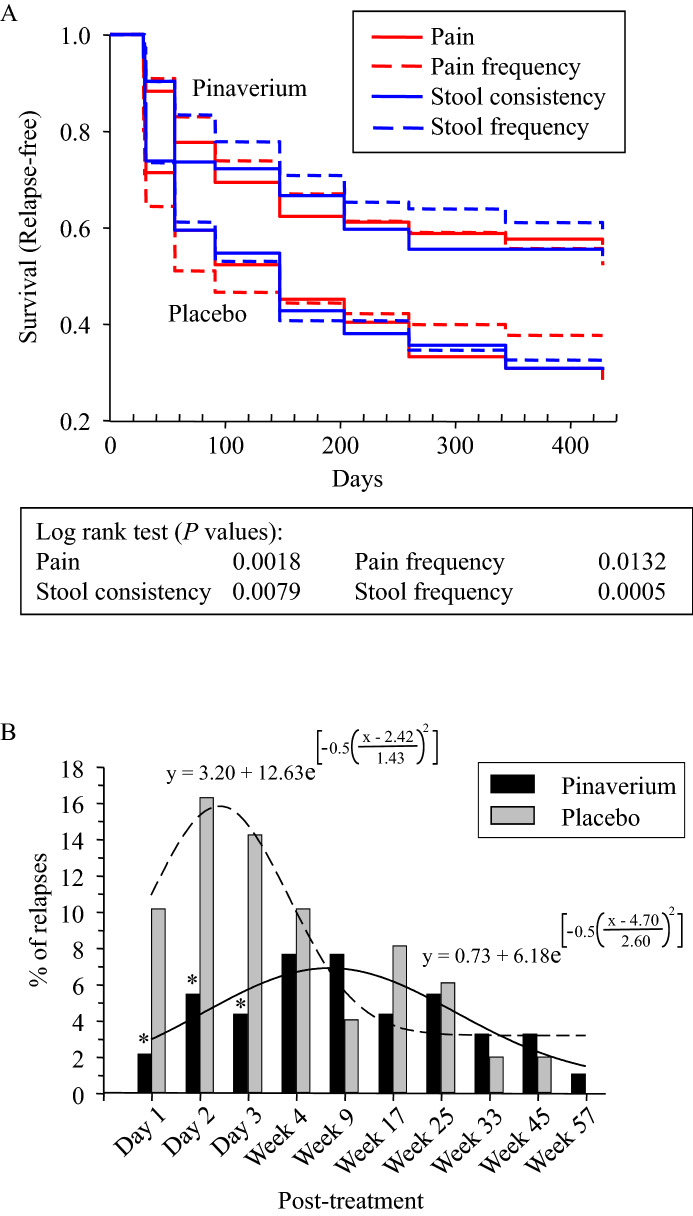
(**A**) Kaplan–Meier analysis on the relapse-free events as measured by the symptomatic endpoints (pinaverium: n = 85, 72, 88, and 72 for pain, stool consistency, pain frequency, and stool frequency, respectively; placebo: n = 42, 42, 45, and 49; see the Supplemental Material). (**B**). Distributions of relapses as measured by global overall symptom scales, which were nonlinearly regressed by Gaussian processes. See the Supplemental Material for details.

Patients whose symptoms were significantly improved at the end of treatment were used for the Kaplan–Meier survival (relapse-free) analysis (see the Supplemental Materials for details; pinaverium: n = 85, 72, 88, and 72 for pain, stool consistency, pain frequency, and stool frequency, respectively; placebo: n = 42, 42, 45, and 49). The relapse-free probabilities of all the primary and secondary endpoints in the pinaverium group were significantly higher than those in the placebo group (Fig. 3A) indicating that the therapeutic effects of pinaverium extended beyond the treatment.

Among the patients whose global overall symptom scales were significantly improved by the end of the treatment (pinaverium n = 91, placebo n = 49), symptoms relapsed in a total of 41 (45.1%) patients in the pinaverium group while 36 (73.5%) patients of the placebo group by week 57 reconfirming that the therapeutic effects of pinaverium extended beyond the treatment (*P* < 0.005, *X*_2_^2^ = 10.4). The PTTE of pinaverium could also be seen in the remarkable distribution differences (Fig. 3B). During first few days after the treatment was discontinued, relatively more relapses occurred in the placebo group than in the pinaverium group.

### Natural history of IBS

Seventeen weeks after the treatments were discontinued, there were no significant differences between pinaverium and placebo in terms of all measurements (Fig. 2, Table 2, Supplemental Material Tables S5–S7, and S9). Therefore, the stage after 17 weeks could be considered as IBS natural history. During this period, an average of 51.5–56.4% of patients (pool pinaverium and placebo data together) had IBS symptoms (pain scale ≥ 3, stool consistency ≥ 5) (Table S9). At the end of this study (week 61), 146 (54.9%) patients had IBS symptoms.

### Tertiary endpoint outcomes

Pinaverium significantly and rapidly reduced the scales of the global overall symptom from day 1 (*P* < 0.05; Figure S1). During the first 3 days, the scales reduced 83.4% of the total decrease reconfirming that pinaverium has a rapid onset of action for relieving IBS symptoms. At the end of the treatment, 68.9% (91 patients) of the pinaverium patients’ global overall symptom scales decreased in contrast to 37.1% (49 patients) in the placebo group (*P* < 0.001, *X*_2_^2^ = 26.8). Pinaverium had a slow offset of action. Three days after pinaverium was discontinued, the global overall symptom rebounded only accounted for 42.3% of the entire rebounded scale. The improved global overall symptom was maintained until 9–17 weeks after pinaverium was discontinued. After week 17, there were no significantly differences between pinaverium and placebo (*P* < 0.05).

Pearson correlation coefficient analysis revealed that the global overall symptom scales were most strongly associated to pain during both the treatment and the post-treatment indicating that patients’ global evaluations were mainly based on the pains they suffered (Table S10, *P* < 0.01).

No severe treatment-emergent adverse event (TEAE) occurred during this study that limited patients’ activities, required medical intervention or required hospitalization. A total of 23 patients in the pinaverium group suffered at least 1 TEAEs while 19 patients in placebo group (Table S11). The adverse event profiles of the 2 groups showed a significant difference (Fisher's exact test, *P* < 0.05), indicating that the adverse events in the pinaverium group were caused by the treatment. Most patients had 1 TEAE. Only four patients in the pinaverium group and 2 patients in the placebo group had 2 TEAEs.

Routine laboratory results, vital signs, physical examinations, and electrocardiograms were unremarkable showing no treatment-related effects.

## Discussion

In this trial, we developed a 3-element criterion to study pinaverium’s PTTE, relapse-free probability, and IBS natural history. *First*, pinaverium effectively relieved IBS symptoms so that the PTTE was justified due to the treatment. *Second*, the follow-up duration of current trial lasted 57 weeks covering both the PTTE period and the natural history thereafter. The natural history is defined as the period in which no significant differences in the outcome endpoints between the treatment group and the placebo group. *Third*, we collected data at an adequate data collection resolution (we collected data 10 times during the follow-up period). Our results showed that the significant differences between the outcomes of pinaverium and placebo disappeared between weeks 9 and 17 after the treatment was discontinued (*P* > 0.05, Fig. 2, Table 2, Supplemental Material Tables S7, S9). We concluded that pinaverium PTTE lasted 9–17 weeks. The stage after week 17 could be considered as IBS natural history.

It is important to cover the IBS natural history when assessing PTTE. *First*, roughly half of patients could recover without any treatment after 1 and 7 years^2^. To distinguish the PTTE from IBS natural history, PTTE studies should collect outcome data during both PTTE period and IBS natural history. *Second*, the percentage of patients with IBS symptoms during IBS natural history is an important clinical parameter for the study populations. In the present study, 51.5–56.4% of patients suffered IBS symptoms during the 40-week natural history (weeks 17–57) (Table S9). Our results were consistent with previous studies that approximately half of the patients had unchanged or aggravated symptoms after 5 years^24^, and “patients with unchanged symptoms (30–50%) and patients whose symptoms had worsened (2–18%)” after 6 years^25^.

In contrast, none of the existing trials met the 3-element criterion. The existing trials drew inaccurate or misleading conclusions regarding the PTTE durations. Critical thinking is needed to correctly interpret the results from these existing studies^26^. For example, Pimentel et al. only followed up with patients for 10 weeks after rifaximin was discontinued, and the rifaximin group still showed significantly improved symptoms at week 10 when the trial ended (*P* < 0.05)^4^. Pimentel et al. concluded, “Rifaximin improves IBS symptoms for up to 10 weeks after the discontinuation of therapy.” This is not accurate because “the significantly improved symptoms” might still last beyond 10 weeks. Accordingly, the Editorial Comment on this study is incomplete (“Over a 10-week follow-up period, the rifaximin recipients reported global improvements in overall symptoms and less bloating more frequently than the placebo recipients”^27^) although this comment is correct by itself. An accurate conclusion would be that rifaximin could improve IBS symptoms for *at least* 10 weeks after the discontinuation of therapy. Indeed, some researchers have already noticed this inaccuracy, and suggested that “further randomized controlled trials with active control conditions and longer-term follow-up are needed to determine the effect of such a [treatment]”^28^, and recently, more trials included post-treatment analyses as an essential part though they did not meet the above 3 element criterion (Table S2, trails in italic).

Nevertheless, the preliminary data from these existing studies suggested the treatment-dependency of PTTE (Table 3). In particular, CBT had the longest PTTE (roughly one year or so), while 5-HT_3_ antagonist, alosetron, the shortest (less than one week), and antidepressants have 4 weeks to 1 year PTTE^7,8^.

**Table 3:** Treatment-dependency of post-treatment therapeutic effects.

Treatment	Post-treatment			References
	Follow-up duration	Date collected at	Did the therapeutic effects last?	
Diet				
Low FODMAP diet	1 wk	wk 1	≥ 1 wk	^29^
Probiotics				
*S. cerevisiae*	3 wk	Every wk	1–2 wk	^30^
*L. plantarum*	12 mo	mo 12	≥ 12 mo	^31^
Antibiotics				
Rifaximin	10 wk^a^	Every wk	≥ 10 wk	^13^
	10 wk	Every wk	≥ 10 wk	^4^
	3 mo	Every wk	≥ 3 mo	^32^
Antispasmodics				
*Phloroglucinol*	*1 wk*	*wk 1*	≥ *1 wk*	^33^
Otilonium	10 wk	wk 3, 6, 10	≥ 10 wk	^15^
pinaverium	57 wk	dy 1, 2, 3, wk 4, 9, 17, 25 ,33, 45, 57	between 9 and 17 wk	Current study
Peppermint oil				
Anise oil	2 wk	wk 2	≥ 2 wk	^34^
Peppermint oil	4 wk	wk 4	≥ 4 wk	^6^
Antidepressants				
Fluoxetine	4 wk	wk 4	≥ 4 wk	^7^
Paroxetine 20 mg, QD	1 yr	yr 1	< 1 yr (very low DCR)	^8^
Psychological therapies				
Cognitive behavior therapy	2 wk	wk 2	≥ 2 wk	^10^
*Mindfulness-based cognitive therapy*	*6 wk*	*wk 6*	≥ *6 wk*	^35^
Cognitive-based tx	3 mo	mo 3	≥ 3 mo	^36^
Cognitive-behavioral group therapy	3 mo	mo 3	≥ 3 mo	^27^
Mindfulness-based stress and pain management	3 mo	After tx, mo 3	≥ 3 mo	^37^
Relaxation response meditation	3 mo	wk 2, mo 3	≥ 3 mo	^38^
Cognitive-behavioral internet therapy	3 mo	wk 6, mo 3	≥ 3 mo	^39^
Cognitive behavioral therapy	3 mo	wk 1, 2, 3, mo 3	≥ 3 mo	^40^
*Telephone/web CBT*	*4 mo*	*mo 4*	≥ *4 mo*	^11^
Behavioral treatment	5 mo	mo 5	≥ 5 mo	^41^
Cognitive behavioral therapy	6 mo	mo 2, 3, 6	≥ 6 mo	^42^
Mindfulness-based stress reduction	6 mo	mo 6	< 6 mo (low DCR)	^28^
Psychodynamic inter-personal therapy	1 yr	yr 1	< 1 yr (very low DCR)	^8^
Hypnotherapy	1 yr	mo 3, yr 1	≥ 1 yr	^43^
Comprehensive self-management	1 yr	mo 3, 6, 12	≥ 1 yr	^11^
Comp CBT: 8 wkly 1 h Brief CBT: one 90 min	12 mo	wk 9, mo 6, 12	≥ 1 yr	^9^
5-HT_3_ antagonist				
Alosetron, 1 mg, BID	2 wk	Every wk	< 1 wk	^44^
	4 wk	Every wk	< 1 wk	^45–47^
	1 mo	mo 1	< 1 mo	^48^

Regular font: The trials reviewed by Ford et al.^5^. Italic font: updated studies not reviewed by the American College of Gastroenterology.

*CBT* cognitive behavior therapy, *Comp* comprehensive, *DCR* data collection resolution; *dy* day(s), *FODMAP* fermentable oligosaccharides, disaccharides, monosaccharides, and polyols; *mo* month(s), *QD* every day, *yr* year.

^a^Repeated treatment design: 2 wk of first rifaximin treatment + 10 wk follow-up + 2 wk of second rifaximin treatment + 4 wk follow-up. Data from the first treatment and the first follow-up were used here.

Heitkemper et al. showed that patients in both 8-week psychotherapy and 1-day psychotherapy showed significant PTTE one year after the psychotherapy (*P* < 0.05)^9^. Lackneret al. showed that both patient-administered CBT and therapist-administered CBT significantly improved IBS symptoms when compared with the control 2 weeks after the 10-week treatments (*P* < 0.05)^10^. Jarrettet al. showed that the psychotherapy was “efficacious whether delivered primarily by telephone or totally in-person” one year after the therapy^11^. Everitt et al. showed that both telephone and web “interventions were superior to TAU (the control group) up to 12 months of follow-up”^11^. All the above evidence reaffirmed that PTTE is “treatment-dependent” regardless of the length of the treatment, who administered the treatment, and how the treatment was administered.

The current trial showed that antispasmodics have 9–17 weeks of PTTE in consistence with previous studies showing that the PTTE of otilonium lasted at least 10 weeks^15^. That the PTTE in current trial with a 4-week treatment had a similar length to that of Clavé et al. with a 15-week treatment^15^ indicated that the PTTE in pharmacological therapies less depends on the length of the treatment like that in non-pharmacological therapies or CBT.

The mechanism of the treatment-dependency of PTTE is likely due to the mechanism of action of the treatment. Otilonium and pinaverium are the first two antispasmodics recommended by ACG to manage IBS^5^, and both antispasmodics are voltage activated L-type calcium channel blockers^49,50^. Clavé et al. proposed, “[d]ue to its lipophylic properties, (otilonium’s) affinity for colonic smooth muscle may extend beyond the treatment period, and this could explain the prolonged efficacy after cessation of drug intake.”^15^ We believe that in addition that the “affinity for colonic smooth muscle may extend beyond the treatment period”, the prolonged efficacy of pinaverium was also due to the improvement of the function of the colonic smooth muscles during the treatment, and this improvement extended beyond the treatment period^50–53^. This hypothesis is consistent with the nature of IBS, a functional disorder, and further supported by the results from Camilleri et al., who showed that the efficacy of alosetron in IBS waned within one week after alosetronwas discontinued^45^. Alosetron reduces IBS symptoms through peripheral antinociception and inhibition of emotional motor system regions in the brain^54^, both of these mechanisms do not primarily improve the colonic function so that the efficacy of alosetron waned quickly after the treatmentwas discontinued. It is interesting to note that, in contrast to other treatments, probiotics showed remarkable heterogeneous results. *S. cerevisiae* has only 1–2 weeks of PTTE while *L. plantarum* has at least 1 year of PTTE^30,31^. This heterogeneity is consistent with the evidence that different probiotics benefit gastrointestinal functions differently^55^.

In addition to not fulfilling the above 3-element criterion, all the existing trials failed to address another important question, how quickly their therapy took effects. In these trials, the soonest outcome data were collected one week after the therapies were initiated. In this present study, data were collected from the second day after pinaverium was initiated. With this data collection resolution, our study demonstrated that the decreases in the outcome scales during the first 3 days of treatment accounted for 75.5–94.5% of the total decreases. The fast onset of action might be because the lipophilic properties of pinaverium render a high affinity for colonic smooth muscle^15^. Our results justified pinaverium being used as a first line rescue medication.

Assessing the onset of action, establishing PTTE, and accurately assessing the length of PTTE are clinically important for efficient long-term IBS management. Our results provide important insights into cost-effective management of refractory IBS. For example, for patients with refractory IBS, a therapy with a longer PTTE should be initiated while for patients who have an urgent need to improve their IBS symptoms, a medication with a fast onset of action is preferred.

In conclusion, the PTTE of IBS therapy is treatment-dependent. The PTTE of pinaverium lasted 9–17 weeks in contrast to ≥ 1 year PTTE in CBT and < 1 week PTTE in 5-HT_3_ antagonist (alosetron) treatment. PTTE length less depends on the treatment length. The selection of a treatment should consider both its effectiveness during treatment and its PTTE after treatment. Pinaverium can be used as a first line rescue medication for a quick symptom relief plus a 9–17 week long PTTE.

## Supplementary Information

**Supplementary Information**.

## Abbreviations

5-HT_3_: 5-Hydroxytryptamine
CBT: Cognitive behavior therapy
IBS: Irritable bowel syndrome
PTTE: Post-treatment therapeutic effects
RR: Relative risk
