# Supplementary material for: Assessing the post-treatment therapeutic effect of pinaverium in irritable bowel syndrome: a randomized controlled trial
Source: Sci Rep. 2021 Jul 6;11:13894. doi: 10.1038/s41598-021-92990-7 (PMC8260803; doi:10.1038/s41598-021-92990-7)
Supplement: Supplementary file 1 — Supplementary Information. [file 41598_2021_92990_MOESM1_ESM.doc]

Assessing the post-treatment therapeutic effect of pinaverium in irritable bowel syndrome: a randomized controlled trial

Liang Zheng^1^, M.D., Weimin Lu^2^, M.D., Qi Xiao^3^, M.D., Yaoliang Lai^4^, M.D., Heng Fan^5^, M.D., Yuling Sun^6^, MS., Dawei Huang^4^, M.D., Yuanyuan Wang^1^, M.D., Zhen Li^1^, M.D., Zhengyan Jiang^1^, M.D., Xingxing Liu^5^, M.D., Lijuan Zhang^5^, M.D., Dongmei Zuo^5^, M.D., Zhexing Shou^5^, M.D., Qing Tang^5^, M.D., Huisuo Huang^7^, Ph.D., Yongqiang Yang^7^, M.S., Zongxiang Tang^6^, Ph.D., and Jun Xiao^7,†^, PharmD, Ph.D.

1: Department of Gastroenterology, the Second Affiliated Hospital of Nanjing University of Chinese Medicine. 23 Nanhu Rd., Nanjing, 210017, China.

2: Department of Internal Medicine, Jiangsu Provincial Hospital of Chinese Medicine, Affiliated Hospital of Nanjing University of Chinese Medicine. 155 Hanzhong Rd., Nanjing, 210029, China.

3: The School of Medicine, Washington University. 660 S Euclid Ave, St. Louis, MO 63110, the United State.

4: Department of Gastroenterology, Beijing Xuanwu Hospital of Chinese Medicine. 8 Wanming Rd., Beijing 100050, China.

5: Department of Integrated Chinese Medicine and Western Medicine, Union Hospital, Tongji Medical College, Huazhong University of Science and Technology. 1277 Liberty Rd., Wuhan, 430022, China.

6: The State Key Laboratory Cultivation Base for TCM Quality and Efficacy, the School of Medicine and Life Sciences, Nanjing University of Chinese Medicine, 138 Xianlin Road, Nanjing 210023, China.

7: The Macrohard Institute of Health. 231 North Ave. Battle Creek, MI 49017, USA.

^†^: Corresponding Authors: Jun Xiao, PharmD., Ph.D. The Macrohard Institute of Health. 231 North Ave. Battle Creek, MI 49017, USA. Email: jxiao@macrohardinstitute.org. Phone: (01) 248-880-5250. Fax: (01) 248-773-7504.

#### On-line Supplemental Material

Survival (Relapse-free) Analysis ……………………………………………………...……………………………… 2

Table S1.  The durations of the treatments and the post-treatments in the clinical

studies reviewed by the American College of Gastroenterology for its recommendations ......………....… 2

Table S2. Studies on post-treatment therapeutic effects……………………………………………......................….. 4

Table S3. Irritable Bowel Syndrome (IBS-D) Questionnaire ..…...........................………………………..........….… 9

Table S4. The Bowel Symptom Scale (BSS) .....……………………….............…………………………………… 10

Table S5. The mean (95% confidence interval) of the primary and secondary endpoints (ITT[[1]](#footnote-2)) ..........…………..… 11

Table S6. The mean (95% confidence interval) of the primary and secondary endpoints (PPError: Reference source not found) …………………….. 12

Table S7. The relative risk (RR) and 95% confidence interval (CI) of the primary and secondary endpoints ………. 13

Table S8. The Average Inter-item Correlation (r) analysis of the primary and secondary endpoints ….……………. 14

Table S9. The number (percentage) of patients with IBS symptoms ...….…………………………………….....….. 16

Table S10. Average Inter-item Correlation (r_∑_) analysis between global overall symptom scales and symptomatic endpoints during / post- treatment (ITT) ........…………………………………………….. 16

Table S11. Patients with at least one treatment-emergent adverse effects (ITT) ………....…………………………..17

Figure S1. The time course of the global overall symptom during the treatment and post-treatment ………………...17

References ……………………………………………………………………………………………………..…...…. 17

##### Survival (Relapse-free) Analysis

The Kaplan-Meier survival curve is one of the most frequently used statistical methods to analyze the probability of disease free over a period of time.[[2]](#endnote-2),[[3]](#endnote-3) In disease free survival curves, the event of interest is relapse of a disease.[[4]](#endnote-4)

There are three assumptions used in this analysis.  First, at any time patients who are censored have the same survival prospects as those who continue to be followed;  second, the survival probabilities are the same for subjects recruited early and late in the study;  third, the event happens at the time specified.[[5]](#endnote-5) This study satisfies these assumptions.

Relapse, per definition, is a deterioration after a period of improvement. Thus, only patients whose symptoms were significantly improved at the end of treatment were used for the Kaplan-Meier survival (relapse-free) analysis (Figure 3A).

Table S1. The durations of the treatments and the post-treatments in the clinical studies reviewed by the American College of Gastroenterology for its recommendations

| Therapies  Recommendation | | # of RCT^4^;  # of patients. | Durations of each studies  (Treatment + Post-treatment) |
| --- | --- | --- | --- |
| Exercise  Recommendation: weak | | 2 RCTs (38,39);  158 patients. | 12W+0; 12W+0. |
| Diet & dietary manipulation | Low FODMAP diet  Recommendation: weak | 7 RCTs (43–49);  397 patients. | 4W+0; 4W+0; 3W+0; *4W+7D*, 4W+4W^5^; 3W+0; *** (a cross-over study). |
|  | Gluten-free diet^1^  Recommendation: weak | 2 RCTs (54, 55);  111 patients. | 6W+0; 6W+0. |
| Fiber  Recommendation: strong | | 15 RCTs (58–72);  946 patients. | 6W+0; 6W+0; 4W+0; 8W+0; 4W+0; no info; 16W+0; 3M+0; 12W+0; 4W+0; 3M+0; 12W+0; 12W+0; 4W+0; 4W+0. |
| Prebiotics^1^  Recommendation: weak | | 1 RCT (80);  128 patients. | 8W+0. |
| Synbiotics^1^  Recommendation: weak | | 2 RCTs (81, 82);  198 patients. | 12W+0; 8W+0. |
| Probiotics  Recommendation: weak | | 37 RCTs (47, 84, 86–89, 91,  92, 94-104, 110, 112, 113,  115, 118, 119, 121, 123,  125-134);  4403 patients. | 4W+0; 8W+2W^5^; 12W+0; 4W+0; 6W+0; 4W+0; 6W+0; *8W+3W*; 4W+0; *8W+8W*; 12W+0; 8W+0; 10W+0; 12W+0; 4W+0; *4W+12M*; 4W+0; 10W+0; 6M+0; 4W+0; 4W+0; 8W+0; 4W+0; 8W+0; 8W+0; 8W+0; 4W+0; 8W+0; 8W+0; 10W+0; 4W+0; 4W+0; 4W+0; *** (two 3-week tx separated by 4 washout); 12W+0; *6M+6M*; 12W+0. |
| Antibiotics (rifaximin)  Recommendation: weak | | 6 RCTs (137, 139–142);  2441 patients. | *2W+10W^6^*; 30D+0; *10D+10W*; ***^7^; *2W+10W (TARGET 1) / 3M (TARGET 2)*. |
| Antispasmodics   Recommendation: weak | | 26 RCTs (60, 63, 64, 149–  171);  2811 patients. | 4W+0; no info; 16W+0; 4W+0; 4W+0; ***^7^; 4W+0; 6M+0; 3M+0; 1M+0; 4W+0; 2W+0, 1W+0; 6M+0; 60D+0; 4W (and then crossed over)+0; no info; 15W+0; 4W+0; 12W+0; 6W+0; 2W+0; 4W+0; ***^8^; 4W+0; *15W+10W*. |
| Peppermint oil | | 7 RCTs (172–178);  634 patients. | 4W+0; *4W+2W*; 4W+0; 1M+0; *4W+4W*; 3M+0; 8W+0. |
| Antidepressants | TCAs  Recommendation: strong | 12 RCTs (63, 188–194, 197,  200, 201, 204);  787 patients. | no info; 10W+0; 2M+0; 4W+0; 8W+0; 3M+0; 6W+0; 12W+0; 2M+0; 12W+0; 12W+0; 2M+0. |
|  | SSRIs   Recommendation: weak | 7 RCTs (195, 196, 198–200,  202, 203);  356 patients. | 6W+0; 7W+0; *12W+4W*; 6W+0; 12W+0; 12W+0; 8W+0. |
| Psychological therapies | | 36 RCTs (published in 34  articles: 194, 211–243);  2487 patients. | 12W+0; 8W+0; 12M+0; *8W+3M*; *2W+3M*; 8W+0; 8W+0; *6W+3M*; *1M+5M*; 12M+0; 8W+0; 12W+0; *** (a crossover study); *12W+3M*; *12W+1Y*; 8W+2Y (no Control -- compared with baseline); *8W (Comprehensive tx) / 1D (Brief tx)+12M*; *8W+0; 8W+0; 7W+6M; 5W+3M; 10W+3M; 6M+0; 3M+1Y; 8W+6M*; *8W+3M*; 2M+0; 10W+0; 10W+0; *10W+2W*; *9W+1Y*; 8W+1Y^9^; 8W+0; 3W+10W. |
| Prosecretory agents | Linaclotide^2^  Recommendation: strong | 4 RCTs (245–248);  2867 patients. | 12W+0; 12W+0; 26W+0; *** (a crossover clinical trial). |
|  | Plecanatide^2^  Recommendation: strong | 3 RCTs (2 RCTs published  in 249, 1 published in 250);  2612 patients. | 12W+0; 12W+0. |
|  | Lubiprostone^2^  Recommendation: strong | 3 RCTs (3 RCTs published  in 254 and 255);  1366 patients. | 12W+0, 3M+0. |
| Eluxadoline^3^  Recommendation: weak | | 3 RCT (3 RCTs published in 257 and 258);  3235 patients. | 52W+0; 12W+0. |
| Loperamide^1^  Recommendation: strong | | 2 RCTs (259, 260);  42 patients. | 3W+0; 13W+0. |
| Alosetron^3^  Recommendation: weak | | 8 RCTs (265–272);  4987 patients. | 12W+0; *12W+4W*; *12W+4W*; 12W+0; *12W+2W*; *48W+4W*; *12W+4W*; 12W+0. |
| Polyethylene glycol^1^ | | 2 RCTs (274,275);  181 patients. | 30D+0, 4W+0. |
| 5-aminosalicylates^1^  Recommendation: weak | | 3 RCTs (281–283);  464 patients. | *12W+12W*; 12W+0; 12W+0. |

This table is the summary of Ford et al. American College of Gastroenterology Monograph on Management of Irritable Bowel Syndrome. Am J Gastroenterol. 2018;113 (Suppl 2):1-18.

Note: 1: not recommended. 2. recommended in IBS-C patients. 3: recommended in IBS-D patients. 4: the reference numbers in this column refer to the references cited by Ford et al. 2018. 5: 2 X 2 factorial trial, not comparing a single treatment with a placebo/control. 6: Repeated treatment design: 2 weeks of first rifaximin treatment + 10 weeks follow-up + 2 weeks of second rifaximin treatment + 4 weeks follow-up. Data from the first treatment and the first follow-up were used here. 7: preliminary results as an Abstract only. 8: this study (Ref. 169, Castiglione et al. 1991) is a review article, not an original research article. 9: this study (Boyce at al. 2003) did not compare a psychological therapy alone with a treatment as usual; this study compared three arms (a. cognitive behavior therapy plus standard care therapy, b. relaxation therapy plus standard care therapy, and c. standard care therapy). This design could not single out the effects of either behavior therapy.

Abbreviation: D = days; M = months; Y = year; no info = no information; SSRI = selective serotonin reuptake inhibitor; TCA = tricyclic antidepressant; tx = treatment; W = weeks.

Italics are randomized, controlled studies with post-treatment follow-up. Details of these studies were summarized in Table S2.

Table S2. Studies on post-treatment therapeutic effects.

| Trail # | Trails | Study design | S / M-sites?^1^ | # of pt (ITT) | | Treatment | | | | | | Post-treatment | | | | | | | | |
| --- | --- | --- | --- | --- | --- | --- | --- | --- | --- | --- | --- | --- | --- | --- | --- | --- | --- | --- | --- | --- |
|  |  |  |  | Tx group | Placebo (Control) group | Tx | Tx Duration | Date collected at | Outcome endpoints | FDA endpnts?^2^ | Tx effective? | Post-tx follow-up duration | Date collected at | + therapeutic effect?^3^ | + PTTE duration | Entire PTTE?^4^ | Know + PTTE duration?^5^ | IBS natural history?^6^ | Studied relapse-free probability? | Pt # (%) stayed relapse-free |
| 1 | Staudacher et al. 2012.[[6]](#endnote-6) | randomized, controlled | S | 19 | 22 (habitual diet) | Low FODMAP diet | 4 wk | wk 1 | bloating, pain, flatulence, borborygmus, urgency, diarrhea, constipation, incomplete evacuation, heartburn, nausea, lethargy. | N | Y | 1 wk | wk 1 | Y | ≥ 1 wk | N | N | N | N | N |
| 2 | Pineton et al. 2015.[[7]](#endnote-7) | double-blind, randomized, placebo-controlled | S | 86 | 93 | S. cerevisiae | 8 wk | every wk. | pain / discomfort; bloating/distension, bowel movement difficulty, stool frequency and consistency. | N | Y | 3 wk | every wk. | Y | 1-2 wk | Y | Y | 1 wk (but not adequate) | N | N |
| 3 | Nobaek et al. 2000.[[8]](#endnote-8) | double-blind, randomized, placebo-controlled | S | 30 | 30 | L. plantarum | 4 wk | every wk | defecation #, fecal consistency, abundant gas, overall GI function, defecation function, pain, and flatulence. | N | Y | 12 mo | mo 12 | Y | ≥ 12 mo | N | N | N | N | N |
| 4 | Begtrup et al. 2013.[[9]](#endnote-9) | double-blind, randomized, placebo-controlled | S | 67 | 64 | probiotics | 6 mo | every mo | pt % of relief; GI symptom changes, QoL. | N | N | 6 mo | every mo | N/A | N/A | N/A | N/A | N/A | N/A | N/A |
| 5 | Lembo et al. 2016.[[10]](#endnote-10) | double-blind, randomized, placebo-controlled | M | 328 | 308 | rifaximin | 2 wk^7^ | every wk | responding % to pain, to loose stool frequency; urgency, global IBS symptoms. | N | Y | 10 wk^7^ | every wk | Y | ≥ 10 wk | N | N | N | N | N |
| 6 | Pimentel et al. 2006.[[11]](#endnote-11) | double-blind, randomized, placebo-controlled | M | 43 | 44 | rifaximin | 10 days | wk 0,1 | pain, diarrhea, constipation, bloating, urgency, incomplete evacuation, mucus, gas. | N | Y | 10 wk | every wk | Y | ≥ 10 wk | N | N | N | N | N |
| 7 | Pimentel et al. 2011.[[12]](#endnote-12) | double-blind, randomized, placebo-controlled | M | 309 / 316 | 314 / 321 | rifaximin | 2 wk | every wk | pt % of relief of global symptoms; pt % of relief of bloating; pt % of global symptoms, bloating, pain, and stool consistency. | N | Y | 3 mo | every wk | Y | ≥ 3 mo. | N | N | N | N | N |
|  |  |  |  | TARGET 1 / TARGET 2 | |  |  |  |  |  |  |  |  |  |  |  |  |  |  |  |
| 8 | Clavé et al. 2011.[[13]](#endnote-13) | double-blind, randomized, placebo-controlled | M | 179 | 177 | otilonium | 15 wk | wk 0, 5, 10, 15. | pain frequency; pt global assess, MD global assess, pain, bloating-meteorism, stool consistency / frequency, mucus; QoL; response rates. | N | Y | 10 wk | wk 3,6,10. | Y | ≥ 10 wk | N | N | N | Y | N |
| 9 | Moaffa et al. 2016.[[14]](#endnote-14) | double-blind, randomized, placebo-controlled | S | 40 | 40 placebo;  40 pepper-mint oil. | anise oil | 4 wk | wk 0, 4 | pain, discomfort, bloating, diarrhea, constipation severity, difficulty in defecation, GI reflux, headache, tiredness, overall satisfaction; QoL. | N | Y | 2 wk | wk 2 | Y | ≥ 2 wk | N | N | N | N | N |
| 10 | Cappello et al. 2007.[[15]](#endnote-15) | double-blind, randomized, placebo-controlled | S | 28 | 29 | peppermint oil | 4 wk | wk 0, 4 | bloating, pain/discomfort, diarrhoea, constipation, incomplete evacuation, gas or mucus and urgency at defecation. | N | Y | 4 wk | wk 4 | Y | ≥ 4 wk | N | N | N | N | N |
| 11 | Vahedi et al. 2005.[[16]](#endnote-16) | double-blind, randomized, placebo-controlled | S | 22 | 22 | fluoxetine | 12 wk | every 2 wk | discomfort, bloating, stool consistency, bowel movement frequency, bowel habit | N | Y | 4 wk | wk 4 | Y | ≥ 4 wk | N | N | N | N | N |
| 12 | Payne & Blanchard. 1995.[[17]](#endnote-17) | randomized, controlled | S | 12 | 10 WLC; 12 self-help support | cognitive-based tx | 8 wk | wk 0, 8 | pain, tenderness, constipation, diarrhea, flatulence, belching, bloating; BDI, STAI; DAS, ATQ. | N | Y | 3 mo | mo 3 | Y | ≥ 3 mo | N | N | N | N | N |
| 13 | Tkachuk et al. 2003.[[18]](#endnote-18) | randomized, controlled | M | 14 | 14 home-based symptom monitor | cognitive-behavioral group therapy | 9 wk | every wk | IBS symptoms (abdominal pain, diarrhea, constipation, bloating, flatulence, and nausea), psychological functioning, QoL. | N | Y | 3 mo | mo 3 | Y | ≥ 3 mo | N | N | N | N | N |
| 14 | Keefer et al. 2001.[[19]](#endnote-19) | randomized, controlled | S | 8 | 8 WLC | relaxation response meditation | 6 wk | wk 0 | primary IBS symptom reduction | N | Y | 3 mo | wk 2, mo 3 | Y | ≥ 3 mo | N | N | N | N | N |
| 15 | Lynch and Zamble. 1989.[[20]](#endnote-20) | randomized, controlled | S | 11 | 10 WLC | behavioral treatment | 8 wk | before and after the tx | pain, disomfort, stool consistency, mucus, bloating, flatulence, belching, nausea, insomnia, anxiety, QoL, bowel movements, analgestics usage, psychotropics usage. | N | Y | 5 mo | mo 5 | Y | ≥ 5 mo | N | N | N | N | N |
| 16 | Lindfors et al. 2012.[[21]](#endnote-21) | randomized, controlled | M | 45 / 25 | 45 support therapy / 23 WLC | hypnotherapy | 12 wk | wk 0 | bloating, gas, pain, loose stools, urgency, hard stools, and incomplete evacuation, QoL. | N | Y | 1 yr | mo 3, yr 1 | Y | ≥ 1 yr | N | N | N | N | N |
|  |  |  |  | Study 1 / Study 2 | |  |  |  |  |  |  |  |  |  |  |  |  |  |  |  |
| 17 | Heitkemper et al. 2004[[22]](#endnote-22) | randomized, controlled | S | Comp: 40 Brief: 48 | 44 TAU | Comp:  8 wkly 1 hr Brief:  one 90 min | Comp: 8 wk Brief: 1 day | wk 0 | pain, bloating, gas, constipation, diarrhea, QoL, SCL-90R, GSI, CSBD. | N | Y | 12 mo | wk 9, mo 6, 12 | Y | ≥ 1 yr | N | N | N | N | N |
| 18 | Moss-Morris et al. 2010.[[23]](#endnote-23) | randomized, controlled | S | 31 (CBT + TAU) | 33 TAU | cognitive behavioral therapy | 7 wk | wk 0 | global assessment, IBS Syndrome Scoring System. | N | Y | 6 mo | mo 2, 3, 6 | Y | ≥ 6 mo | N | N | N | N | N |
| 19 | Hunt et al. 2009.[[24]](#endnote-24) | randomized, controlled | via internet | 28 | 26 WLC | cognitive-behavioral internet therapy | 5 wk | wk 0 | bloating, diarrhea, constipation, pain, satiety; Anxiety Sensitivity Index; Visceral anxiety sensitivity; QoL; Consequences of Physical Sensations Questionnaire. | N | Y | 3 mo | wk 6, mo 3 | Y | ≥ 3 mo | N | N | N | N | N |
| 20 | Ljotsson et al. 2010.[[25]](#endnote-25) | randomized, controlled | via internet | 43 | 43 WLC | cognitive behavioral therapy | 10 wk | every wk | pain, tenderness, diarrhea, constipation, bloating, flatulence, belching and nausea; QoL, GI-specific anxiety, depression, functioning. | N | Y | 3 mo | wk 1, 2, 3, mo 3 | Y | ≥ 3 mo | N | N | N | N | N |
| 21 | Creed et al. 2003[[26]](#endnote-26) | single-blind, randomized, controlled | M | 85  psycho-therapy;  86  paroxt-ine. | 86 tx as usual | Group 1: psycho-therapy; Group 2: paroxetine 20 mg, QD | 3 mo | mo 0, 3 | pain, # of days with pain, overall symptoms. | N | Y | 1 yr | yr 1 | no conclusion  due to low DCR | < 1 yr (very low DCR) | Y | N | Y | N | N |
| 22 | Zernicke et al. 2013.[[27]](#endnote-27) | randomized, controlled | S | 43 | 47 WLC | mindfulness-based stress reduction | 8 wk | wk 0, 8 | pain, pain frequency, distension, bowel habit dissatisfaction, and life interference; QoL; stress, mood, and spirituality scales. | N | Y | 6 mo | mo 6 | Y | < 6 mo (low DCR) | Y | N | N | N | N |
| 23 | Gaylord et al. 2011.[[28]](#endnote-28) | randomized, controlled | S | 36 | 39 social-support | mindfulness-based stress and pain management | 8 wk | wk 0 | IBS severity scale; QoL, visceral sensitivity index, brief symptom inventory-18. | N | Y | 3 mo | after tx, mo 3. | Y | ≥ 3 mo | N | N | N | N | N |
| 24 | Lackner et al. 2008.[[29]](#endnote-29) | randomized, controlled | S | 25 pt CBT^8^:  23 tpt CBT^8^: | 27 WLC | cognitive behavior therapy | 10 wk | wk 0. | relief (pain, bowel symptoms); global symptoms; symptom scale; QoL; psychological distress; pt satisfaction scale. | N | Y | 2 wk | wk 2 | Y | ≥ 2 wk | N | N | N | N | N |
| 25 | Jarrett et al. 2009.[[30]](#endnote-30) | randomized, controlled | S | CSM i/p: 58; t,i/p: 58 | 60 TAU | comp self-management (CSM) | 9 wk | wk 0. | pain, discomfort, bloating, constipation, diarrhea, intestinal gas, urgency, QoL; psychological distress sales. | N | Y | 1 yr | mo 3, 6, 12. | Y | ≥ 1 yr | N | N | N | Y | N |
| 26 | Camilleri et al. 2000.[[31]](#endnote-31) | randomized, placebo-controlled | M | 324 | 323 | alosetron,  1 mg, BID | 12 wk | every wk | relief of pain and discomfort; urgency, stool frequency, stool consistency. | N | Y | 4 wk | every wk | N | < 1 wk | Y | Y | Y (but not adequate) | N | N |
| 27 | Camilleri et al. 2001.[[32]](#endnote-32) | double-blind, randomized, placebo-controlled | M | 309 | 317 | alosetron,  1 mg, BID | 12 wk | every wk | relief of pain and discomfort; urgency, stool frequency, stool consistency, incomplete evacuation, and bloating. | N | Y | 4 wk | every wk | N | < 1 wk | Y | Y | Y (but not adequate) | N | N |
| 28 | Lembo et al. 2001.[[33]](#endnote-33) | double-blind, randomized, placebo-controlled | M | 532 | 269 | alosetron, 1 mg, BID | 12 wk | every wk | day % of satisfactory bowel urgency; global symptoms, stool frequency / consistency, incomplete evacuation. | N | Y | 2 wk | every wk | N | < 1 wk | Y | Y | N | N | N |
| 29 | Chey et al. 2004.[[34]](#endnote-34) | double-blind, randomized, placebo-controlled | S | 351 | 363 | alosetron,  1 mg, BID | 12 mo | every mo | relief of pain and discomfort; urgency, stool frequency, stool consistency, and bloating. | N | Y | 1 mo | mo 1 | N | < 1 mo | Y | Y | N | N | N |
| 30 | Chang et al. 2005.[[35]](#endnote-35) | randomized, double blind, placebo-controlled | M | 127/131/136/ 140 (.5/1/2/4 mg) | 128 | alosetron 0.5, 1.0, 2.0, 4.0 mg, BID | 12 wk | every wk for wk 5 -12 | adequate relief of pain, discomfort; bowel urgency, stool frequency, stool consistency, incomplete evacuation, bloating. | N | Y only for 1 mg | 4 wk | every wk | N | < 1 wk | Y | Y | N | N | N |
| 31 | Barbara et al. 2016.[[36]](#endnote-36) | double-blind, randomized, placebo-controlled | M | 88 | 92 | mesalazine,  800 mg, TID | 12 wk | every wk | relief of pain/discomfort; overall symptoms, bloating, general well-being, stool frequency / consistency, QoL. | N | N | 12 wk | every 4 wk | N/A | N/A | N/A | N/A | N/A | N/A | N/A |
| *32* | *Henrich et al. 2020.[[37]](#endnote-37)* | *randomized, controlled* | *S* | *36* | *31 WLC* | *mindfulness-based cognitive therapy* | *6 wk* | *wk 0,6* | *IBS symptom severity, IBS quality of life, maladaptive catastrophizing, visceral anxiety sensitivity, mindfulness.* | *N* | *Y* | *6 wk* | *wk 6* | *Y* | *≥ 6 wk* | *N* | *N* | *N* | *N* | *N* |
| *33* | *Shin et al. 2020.[[38]](#endnote-38)* | *double-blind, randomized, placebo-controlled* | *S* | *36* | *36* | *phloroglucinol* | *2 wk* | *wk 0,2* | *pain/discomfort, loose/watery stool, urgency, mucus, bloating, gas; QoL.* | *N* | *Y* | *1 wk* | *wk 1* | *Y* | *≥ 1 wk* | *N* | *N* | *N* | *N* | *N* |
| *34* | *Everitt et al. 2019.[[39]](#endnote-39)* | *randomized, controlled* | *M* | *TCBT: 186; WCBT: 185* | *187 TAU* | *telephone CBT (TCBT); web CBT (WCBT)* | *8 mo* | *mo 0,3,6* | *pain, distension/tightness, bowel habit, QoL; Hospital Anxiety and Depression Scale.* | *N* | *Y* | *4 mo* | *mo 4* | *Y* | *≥ 4 mo* | *N* | *N* | *N* | *N* | *N* |

Note: Regular font: the trials reviewed by Ford et al. American College of Gastroenterology Monograph on Management of Irritable Bowel Syndrome. Am J Gastroenterol. 2018;113 (Suppl 2):1-18. Italic font: updated studies not reviewed by the American College of Gastroenterology.

1: Single (S) or multi (M) centers? Unless it was said to be of multi-center, single-center is assumed here.

2: Did the study use FDA recommended endpoints? US Food and Drug Administration. Guidance for industry irritable bowel syndrome — clinical evaluation of drugs for treatment. Final Guidance. Silver Springs, MD: Office of Communications, Division of Drug Information, Center for Drug Evaluation and Research, Food and Drug Administration, 2012.

3: Were there positive post-tx therapeutic effects? As long as at either pain or stool outcome endpoint remained positive post-treatment therapeutic effects, the treatment was considered to have a positive post-treatment effect.

4: Was the post-tx follow-up long enough to reach the end of the extended + therapeutic effects during the post-treatment period?

5: Was the post-tx follow-up long enough and data collection resolution high enough to draw a conclusion on how long + therapeutic effects could last?

6: Was the post-tx follow-up long enough to cover both the + therapeutic effects period and IBS natural history?

7: Repeated treatment design: 2 wk of first rifaximin treatment + 10 wk follow-up + 2 wk of second rifaximin treatment + 4 wk follow-up. Data from the first treatment and the first follow-up were used here.

8: pt CBT = patient administered CBT or minimal contact CBT; tpt CBT = therapist administered CBT or standard CBT.

**Abbreviations**: assess = assessment; ATQ = the Automatic Thoughts Questionnaire - / +; BDI = the Beck Depression Inventory; CBT = cognitive behavior therapy; Comp = Comprehensive; DAS = the Dysfunctional Attitude Scale; DCR = data collection resolution; FDA = the Food and Drug Administration; FFMQ = the Five Factor Mindfulness Questionnaire; FODMAP = fermentable oligosaccharides, disaccharides, monosaccharides, and polyols; GI = gastrointestinal; HAD = the Hospital Anxiety and Depression; hr = hour; i/p = in person; t,i/p = telephone and in person; ITT = intention to treat; LcS = Lactobacillus casei Shirota; MD = physician; min = minute; mo = month(s); PHQ-12 = the Personal Health Questionnaire-12; plab = placebo; pt = patient; PTTE = post-treatment therapeutic effects; QoL = quality of life; SMTC = symptom monitoring with wkly telephone contact; STAI = the State Trait Anxiety Inventory; TAU = tx as usual; tpt = therapist; tx = treatment; wk = week(s); WLC = wait list control; yr = year.

#### Table S3. Irritable Bowel Syndrome (IBS-D) questionnaire

Patient ID: .

Name: Gander:  M  F Age Phone .

Address ZIP . Others .

| **Treatment history of IBS:**   1. How long have you suffered the symptoms of IBS-D?   ______ years and ______months.   1. Have you ever received treatment?    Yes (go to next Question);   No (go to Question 6)   1. Name of the drug(s) used for IBS?   ___________________________________________________________________________   1. Are you still taking the drugs in Question 3?    Yes → Stop. Need 10 days of drug free   No → How long stop the drug(s)?__________   1. Reasons of stopping the drugs:    Effective, but IBS recurs later;   Effective due to side effects;   Effective but the drugs are expensive;   Somewhat effective, not completely relieve the symptoms;   Not effective;   Others . |
| --- |

| **Diagnostic Criteria of IBS** (*symptom onset at least 6 months prior to diagnosis, and* *in the last 3 months associated with two or more in 7*):   1. How many months do you have recurrent abdominal pain on average at least 1 day/week?   *(Inclusion criteria: in the last 3 months)*   1.  Related to defecation;    Associated with a change in the frequency of stool;   Associated with a change in the form (appearance) of stool  *(Inclusion criteria: two or more of the above)* |
| --- |

## Table S4. The bowel symptom scale questionnaire

**The** **bowel symptom scale questionnaire for follow-up** Patient ID: .


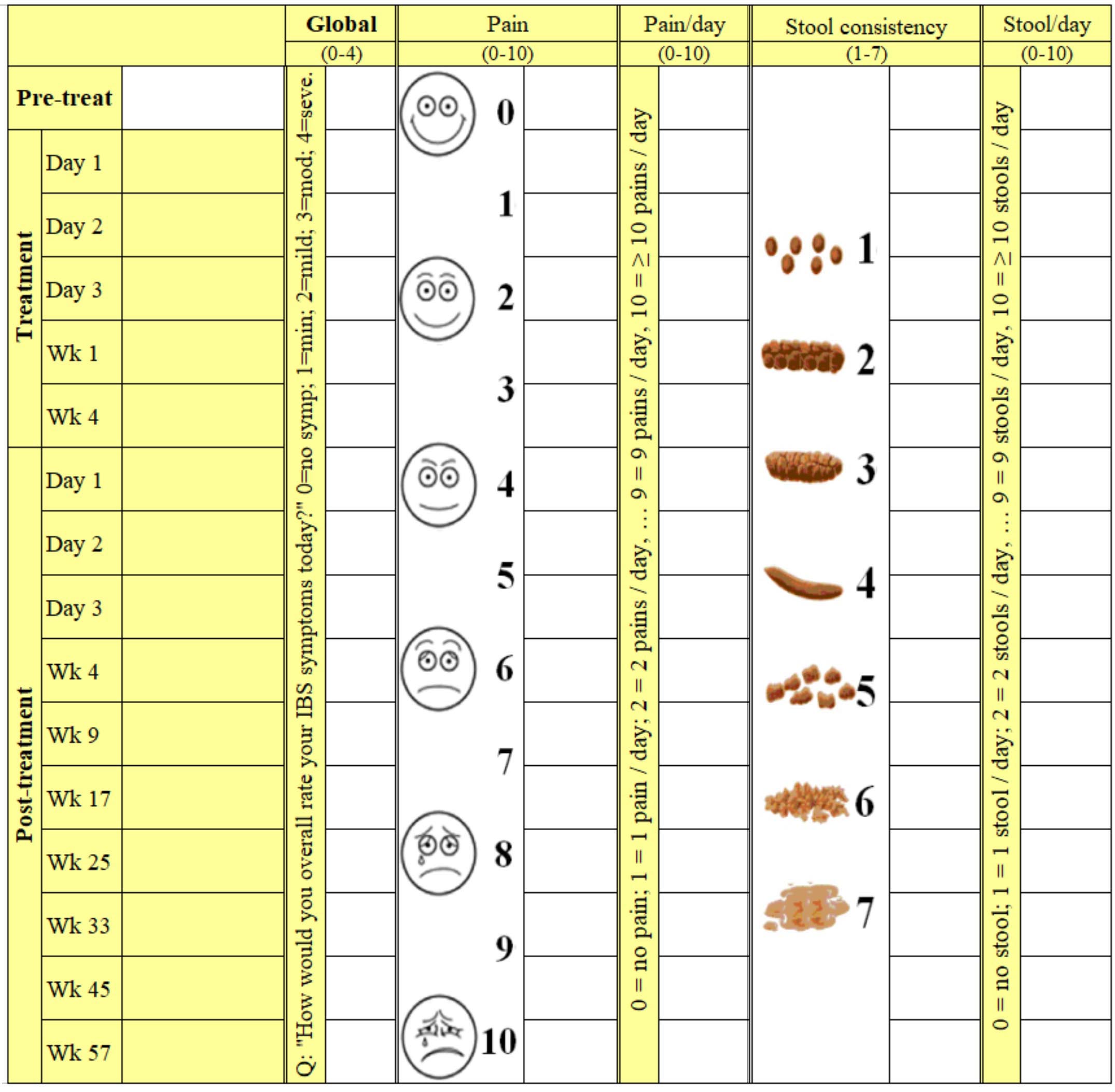


Abbreviation: disc = discomfort; mild = mild symptoms; min = minimum symptoms; mod = moderate symptoms; seve = severe symptoms; Wk = Week.

Table S5. The mean (95% confidence interval) of the primary and secondary endpoints (ITT)

|  | Pinaverium | | | | | | |  | Placebo | | | | | | |  |
| --- | --- | --- | --- | --- | --- | --- | --- | --- | --- | --- | --- | --- | --- | --- | --- | --- |
|  | Primary endpoints | | |  | Secondary endpoints | | |  | Primary endpoints | | |  | Secondary endpoints | | |  |
|  | Pain |  | Stool consist. |  | Pain freq. |  | Stool freq. |  | Pain |  | Stool consist. |  | Pain freq. |  | Stool freq. |  |
|  |  |  |  |  |  |  |  |  |  |  |  |  |  |  |  |  |
| **Treatment** | | | | | | | | | | | | | | | | |
|  | | | | | | | | | | | | | | | | |
| Baseline | 4.92 (4.67–5.18) |  | 6.10 (5.96–6.24) |  | 4.66 (4.40–4.92) |  | 4.36 (4.17–4.54) |  | 5.03 (4.75–5.31) |  | 6.01 (5.96–6.24) |  | 4.56 (4.30–4.82) |  | 4.15 (3.97–4.34) |  |
|  | | | | | | | | | | | | | | | | |
| Day 1 | 3.51 (3.27–3.74) |  | 5.14 (4.92–5.35) |  | 3.28 (3.05–3.51) |  | 3.50 (3.31–3.69) |  | 4.35 (4.11–4.58) |  | 5.53 (4.92–5.35) |  | 4.05 (3.81–4.28) |  | 3.72 (3.54–3.90) |  |
|  | | | | | | | | | | | | | | | | |
| Day 2 | 3.52 (3.25–3.78) |  | 5.08 (4.89–5.27) |  | 3.30 (3.07–3.54) |  | 3.26 (3.08–3.44) |  | 4.24 (4.01–4.48) |  | 5.47 (4.89–5.27) |  | 3.89 (3.66–4.13) |  | 3.60 (3.42–3.77) |  |
|  | | | | | | | | | | | | | | | | |
| Day 3 | 3.20 (2.93–3.47) |  | 4.77 (4.56–4.97) |  | 2.95 (2.71–3.20) |  | 2.91 (2.72–3.10) |  | 4.12 (3.88–4.37) |  | 5.45 (4.56–4.97) |  | 4.02 (3.77–4.27) |  | 3.35 (3.18–3.52) |  |
|  | | | | | | | | | | | | | | | | |
| Week 1 | 3.23 (2.95–3.52) |  | 4.58 (4.35–4.80) |  | 3.00 (2.75–3.25) |  | 2.73 (2.52–2.95) |  | 4.21 (3.95–4.47) |  | 5.22 (4.35–4.80) |  | 3.92 (3.66–4.19) |  | 3.05 (2.85–3.24) |  |
|  | | | | | | | | | | | | | | | | |
| Week 4 | 2.98 (2.67–3.29) |  | 4.33 (4.09–4.57) |  | 2.86 (2.59–3.12) |  | 2.49 (2.28–2.70) |  | 4.06 (3.81–4.31) |  | 5.01 (4.09–4.57) |  | 3.53 (3.26–3.80) |  | 2.90 (2.70–3.10) |  |
|  | | | | | | | | | | | | | | | | |
| **Post-treatment** | | | | | | | | | | | | | | | | |
|  | | | | | | | | | | | | | | | | |
| Day 1 | 3.52 (3.19–3.85) |  | 4.39 (4.14–4.65) |  | 3.11 (2.83–3.39) |  | 2.52 (2.34–2.69) |  | 4.24 (3.99–4.50) |  | 5.02 (4.14–4.65) |  | 3.75 (3.50–4.00) |  | 2.99 (2.82–3.16) |  |
|  | | | | | | | | | | | | | | | | |
| Day 2 | 3.52 (3.17–3.86) |  | 4.49 (4.25–4.73) |  | 3.04 (2.75–3.33) |  | 2.66 (2.46–2.86) |  | 4.37 (4.09–4.65) |  | 5.25 (4.25–4.73) |  | 3.75 (3.52–3.98) |  | 2.94 (2.76–3.11) |  |
|  | | | | | | | | | | | | | | | | |
| Day 3 | 3.63 (3.28–3.98) |  | 4.74 (4.49–4.99) |  | 3.12 (2.82–3.42) |  | 2.92 (2.72–3.13) |  | 4.18 (3.92–4.45) |  | 5.20 (4.49–4.99) |  | 3.82 (3.55–4.08) |  | 3.24 (3.06–3.42) |  |
|  | | | | | | | | | | | | | | | | |
| Week 4 | 3.77 (3.41–4.12) |  | 4.85 (4.60–5.09) |  | 3.45 (3.13–3.78) |  | 3.02 (2.82–3.23) |  | 4.39 (4.09–4.70) |  | 5.27 (4.60–5.09) |  | 3.94 (3.69–4.19) |  | 3.52 (3.34–3.71) |  |
|  | | | | | | | | | | | | | | | | |
| Week 9 | 3.92 (3.57–4.27) |  | 4.88 (4.64–5.12) |  | 3.53 (3.22–3.84) |  | 3.23 (3.03–3.43) |  | 4.44 (4.14–4.74) |  | 5.24 (4.64–5.12) |  | 3.98 (3.70–4.27) |  | 3.61 (3.40–3.83) |  |
|  | | | | | | | | | | | | | | | | |
| Week 17 | 4.00 (3.64–4.36) |  | 4.77 (4.52–5.03) |  | 3.92 (3.56–4.27) |  | 3.22 (3.01–3.43) |  | 4.32 (4.03–4.60) |  | 5.07 (4.52–5.03) |  | 4.12 (3.83–4.41) |  | 3.46 (3.25–3.68) |  |
|  | | | | | | | | | | | | | | | | |
| Week 25 | 4.46 (4.06–4.87) |  | 5.00 (4.76–5.24) |  | 3.66 (3.35–3.97) |  | 3.40 (3.21–3.60) |  | 4.44 (4.11–4.77) |  | 5.02 (4.76–5.24) |  | 4.01 (3.72–4.30) |  | 3.52 (3.30–3.73) |  |
|  | | | | | | | | | | | | | | | | |
| Week 33 | 4.63 (4.23–5.03) |  | 5.08 (4.83–5.32) |  | 3.79 (3.48–4.09) |  | 3.55 (3.33–3.76) |  | 4.47 (4.14–4.80) |  | 5.26 (4.83–5.32) |  | 3.99 (3.70–4.29) |  | 3.41 (3.17–3.65) |  |
|  | | | | | | | | | | | | | | | | |
| Week 45 | 4.58 (4.16–5.01) |  | 4.95 (4.69–5.21) |  | 3.90 (3.56–4.24) |  | 3.32 (3.13–3.51) |  | 4.14 (3.82–4.46) |  | 5.21 (4.69–5.21) |  | 4.20 (3.90–4.50) |  | 3.57 (3.34–3.80) |  |
|  | | | | | | | | | | | | | | | | |
| Week 57 | 4.50 (4.08–4.92) |  | 5.33 (5.06–5.59) |  | 4.00 (3.65–4.35) |  | 3.62 (3.41–3.83) |  | 4.63 (4.30–4.95) |  | 5.05 (5.06–5.59) |  | 3.91 (3.61–4.21) |  | 3.37 (3.15–3.59) |  |
|  | | | | | | | | | | | | | | | | |

Abbreviations: freq. = frequency; Stool consist. = Stool consistency.

Table S6. The mean and 95% confidence interval (CI) of the primary and secondary endpoints (PP)

|  | Pinaverium | | | | | | |  | Placebo | | | | | | |  |
| --- | --- | --- | --- | --- | --- | --- | --- | --- | --- | --- | --- | --- | --- | --- | --- | --- |
|  | Primary endpoints | | |  | Secondary endpoints | | |  | Primary endpoints | | |  | Secondary endpoints | | |  |
|  | Pain |  | Stool consist. |  | Pain freq. |  | Stool freq. |  | Pain |  | Stool consist. |  | Pain freq. |  | Stool freq. |  |
|  |  |  |  |  |  |  |  |  |  |  |  |  |  |  |  |  |
| **Treatment** | | | | | | | | | | | | | | | | |
|  | | | | | | | | | | | | | | | | |
| Baseline | 5.01 (4.71–5.32) |  | 6.09 (5.92–6.25) |  | 4.79 (4.49–5.09) |  | 4.40 (4.19–4.61) |  | 5.14 (4.80–5.48) |  | 6.00 (5.84–6.16) |  | 4.70 (4.38–5.02) |  | 4.17 (3.93–4.41) |  |
|  | | | | | | | | | | | | | | | | |
| Day 1 | 3.61 (3.32–3.90) |  | 5.12 (4.87–5.37) |  | 3.42 (3.16–3.69) |  | 3.50 (3.28–3.72) |  | 4.46 (4.18–4.74) |  | 5.62 (5.44–5.80) |  | 4.09 (3.82–4.36) |  | 3.66 (3.46–3.85) |  |
|  | | | | | | | | | | | | | | | | |
| Day 2 | 3.66 (3.35–3.98) |  | 5.11 (4.89–5.33) |  | 3.39 (3.11–3.67) |  | 3.25 (3.05–3.45) |  | 4.21 (3.91–4.50) |  | 5.49 (5.31–5.68) |  | 3.98 (3.69–4.26) |  | 3.53 (3.32–3.73) |  |
|  | | | | | | | | | | | | | | | | |
| Day 3 | 3.27 (2.95–3.60) |  | 4.76 (4.52–5.00) |  | 3.12 (2.82–3.42) |  | 2.98 (2.76–3.20) |  | 4.21 (3.90–4.21) |  | 5.48 (5.29–5.68) |  | 4.09 (3.78–4.41) |  | 3.33 (3.12–3.33) |  |
|  | | | | | | | | | | | | | | | | |
| Week 1 | 3.32 (2.98–3.66) |  | 4.54 (4.28–4.81) |  | 3.11 (2.81–3.40) |  | 2.62 (2.37–2.87) |  | 4.29 (3.96–4.62) |  | 5.30 (5.04–5.56) |  | 3.93 (3.60–4.27) |  | 3.08 (2.84–3.08) |  |
|  | | | | | | | | | | | | | | | | |
| Week 4 | 3.01 (2.62–3.40) |  | 4.34 (4.05–4.62) |  | 2.89 (2.56–3.22) |  | 2.47 (2.22–2.72) |  | 4.09 (3.78–4.41) |  | 5.07 (4.78–5.36) |  | 3.55 (3.21–3.90) |  | 2.95 (2.70–3.21) |  |
|  | | | | | | | | | | | | | | | | |
| **Post-treatment** | | | | | | | | | | | | | | | | |
|  | | | | | | | | | | | | | | | | |
| Day 1 | 3.62 (3.27–3.97) |  | 4.46 (4.15–4.77) |  | 3.16 (2.86–3.47) |  | 2.51 (2.31–2.71) |  | 4.28 (3.95–4.60) |  | 5.08 (4.80–5.36) |  | 3.75 (3.44–4.05) |  | 2.95 (2.75–3.16) |  |
|  | | | | | | | | | | | | | | | | |
| Day 2 | 3.57 (3.16–3.98) |  | 4.52 (4.23–4.81) |  | 3.12 (2.78–3.46) |  | 2.59 (2.35–2.82) |  | 4.44 (4.09–4.79) |  | 5.24 (5.00–5.48) |  | 3.75 (3.47–4.02) |  | 2.91 (2.69–3.12) |  |
|  | | | | | | | | | | | | | | | | |
| Day 3 | 3.71 (3.28–4.14) |  | 4.77 (4.47–5.07) |  | 3.12 (2.76–3.48) |  | 2.86 (2.61–3.11) |  | 4.28 (3.96–4.60) |  | 5.22 (4.94–5.49) |  | 3.84 (3.51–4.17) |  | 3.17 (2.95–3.40) |  |
|  | | | | | | | | | | | | | | | | |
| Week 4 | 3.86 (3.44–4.28) |  | 4.93 (4.65–5.22) |  | 3.49 (3.11–3.86) |  | 2.98 (2.74–3.22) |  | 4.49 (4.11–4.88) |  | 5.28 (4.99–5.56) |  | 4.01 (3.70–4.32) |  | 3.44 (3.20–3.67) |  |
|  | | | | | | | | | | | | | | | | |
| Week 9 | 3.96 (3.54–4.37) |  | 4.89 (4.60–5.18) |  | 3.63 (3.25–4.01) |  | 3.28 (3.05–3.51) |  | 4.49 (4.11–4.49) |  | 5.31 (5.04–5.58) |  | 4.03 (3.68–4.39) |  | 3.56 (3.30–3.83) |  |
|  | | | | | | | | | | | | | | | | |
| Week 17 | 3.97 (3.55–4.39) |  | 4.85 (4.55–5.15) |  | 3.98 (3.54–4.41) |  | 3.15 (2.90–3.40) |  | 4.39 (4.05–4.73) |  | 5.13 (4.83–5.42) |  | 4.10 (3.75–4.46) |  | 3.48 (3.21–7.75) |  |
|  | | | | | | | | | | | | | | | | |
| Week 25 | 4.51 (4.50–4.97) |  | 4.97 (4.69–5.25) |  | 3.73 (3.37–4.09) |  | 3.33 (3.09–3.56) |  | 4.52 (4.11–4.92) |  | 5.05 (4.76–5.33) |  | 4.02 (3.65–4.40) |  | 3.47 (3.19–3.76) |  |
|  | | | | | | | | | | | | | | | | |
| Week 33 | 4.64 (4.17–5.11) |  | 5.13 (4.83–5.43) |  | 3.84 (3.49–4.18) |  | 3.47 (3.21–3.73) |  | 4.55 (4.16–4.95) |  | 5.24 (4.93–5.55) |  | 4.02 (3.65–4.39) |  | 3.37 (3.05–3.37) |  |
|  | | | | | | | | | | | | | | | | |
| Week 45 | 4.66 (4.16–5.16) |  | 4.95 (4.63–5.26) |  | 4.02 (3.62–4.43) |  | 3.38 (3.14–4.62) |  | 4.20 (3.82–4.57) |  | 5.17 (4.88–5.46) |  | 4.30 (3.95–4.65) |  | 3.55 (3.26–3.84) |  |
|  | | | | | | | | | | | | | | | | |
| Week 57 | 4.55 (4.07–5.04) |  | 5.33 (5.01–5.65) |  | 4.13 (3.72–4.54) |  | 3.62 (3.38–3.86) |  | 4.67 (4.27–5.07) |  | 5.00 (4.69–5.31) |  | 4.00 (3.64–4.36) |  | 3.24 (2.96–3.52) |  |
|  | | | | | | | | | | | | | | | | |

Abbreviations: freq. = frequency; Stool consist. = Stool consistency.

Table S7. The relative risk (RR) and 95% confidence interval (CI) in patients who suffered moderate or severe IBS symptoms (ITT)

|  | Primary endpoints | | | | | | | | | | |  | Secondary endpoints | | | | | | | | | | |  |
| --- | --- | --- | --- | --- | --- | --- | --- | --- | --- | --- | --- | --- | --- | --- | --- | --- | --- | --- | --- | --- | --- | --- | --- | --- |
|  | Pain | | | | |  | Stool consistency | | | | |  | Pain freq. | | | | |  | Stool freq. | | | | |  |
|  | RR | 95% Cl | | | *P* |  | RR | 95% Cl | | | *P* |  | RR | 95% Cl | | | *P* |  | RR | 95% Cl | | | *P* |  |
| **Treatment** | | | | | | | | | | | | | | | | | | | | | | | | |
|  | | | | | | | | | | | | | | | | | | | | | | | | |
| Day 1 | 0.74 | 0.66 | – | 0.83 | < .001 |  | 0.82 | 0.72 | – | 0.93 | < .005 |  | 0.78 | 0.69 | – | 0.89 | < .001 |  | 0.91 | 0.83 | – | 0.99 | < .05 |  |
|  | | | | | | | | | | | | | | | | | | | | | | | | |
| Day 2 | 0.74 | 0.65 | – | 0.84 | < .001 |  | 0.81 | 0.70 | – | 0.92 | < .005 |  | 0.75 | 0.65 | – | 0.87 | < .001 |  | 0.89 | 0.80 | – | 0.99 | < .05 |  |
|  | | | | | | | | | | | | | | | | | | | | | | | | |
| Day 3 | 0.70 | 0.61 | – | 0.81 | < .001 |  | 0.74 | 0.63 | – | 0.86 | < .001 |  | 0.65 | 0.55 | – | 0.76 | < .001 |  | 0.80 | 0.70 | – | 0.92 | < .001 |  |
|  | | | | | | | | | | | | | | | | | | | | | | | | |
| Week 1 | 0.70 | 0.60 | – | 0.81 | < .001 |  | 0.73 | 0.60 | – | 0.89 | < .005 |  | 0.79 | 0.67 | – | 0.91 | < .005 |  | 0.81 | 0.68 | – | 0.97 | < .05 |  |
|  | | | | | | | | | | | | | | | | | | | | | | | | |
| Week 4 | 0.61 | 0.51 | – | 0.72 | < .001 |  | 0.67 | 0.53 | – | 0.83 | < .001 |  | 0.76 | 0.64 | – | 0.91 | < .005 |  | 0.78 | 0.64 | – | 0.95 | < .05 |  |
|  | | | | | | | | | | | | | | | | | | | | | | | | |
| **Post-treatment** | | | | | | | | | | | | | | | | | | | | | | | | |
|  | | | | | | | | | | | | | | | | | | | | | | | | |
| Day 1 | 0.71 | 0.61 | – | 0.81 | < .001 |  | 0.60 | 0.48 | – | 0.76 | < .001 |  | 0.75 | 0.64 | – | 0.88 | < .001 |  | 0.71 | 0.58 | – | 0.87 | < .001 |  |
|  | | | | | | | | | | | | | | | | | | | | | | | | |
| Day 2 | 0.68 | 0.59 | – | 0.79 | < .001 |  | 0.61 | 0.48 | – | 0.76 | < .001 |  | 0.71 | 0.61 | – | 0.83 | < .001 |  | 0.77 | 0.63 | – | 0.94 | < .010 |  |
|  | | | | | | | | | | | | | | | | | | | | | | | | |
| Day 3 | 0.75 | 0.65 | – | 0.87 | < .001 |  | 0.66 | 0.53 | – | 0.82 | < .001 |  | 0.75 | 0.64 | – | 0.88 | < .001 |  | 0.83 | 0.71 | – | 0.96 | < .05 |  |
|  | | | | | | | | | | | | | | | | | | | | | | | | |
| Week 4 | 0.84 | 0.73 | – | 0.97 | < .05 |  | 0.80 | 0.66 | – | 0.98 | < .05 |  | 0.79 | 0.68 | – | 0.91 | < .005 |  | 0.84 | 0.73 | – | 0.98 | < .05 |  |
|  | | | | | | | | | | | | | | | | | | | | | | | | |
| Week 9 | 0.84 | 0.73 | – | 0.97 | < .05 |  | 0.80 | 0.64 | – | 0.99 | < .05 |  | 0.86 | 0.74 | – | 0.99 | < .05 |  | 0.84 | 0.72 | – | 0.99 | < .05 |  |
|  | | | | | | | | | | | | | | | | | | | | | | | | |
| Week 17 | 0.90 | 0.78 | – | 1.03 | > .05 |  | 0.83 | 0.68 | – | 1.02 | > .05 |  | 0.89 | 0.77 | – | 1.02 | > .05 |  | 0.97 | 0.83 | – | 1.13 | > .05 |  |
|  | | | | | | | | | | | | | | | | | | | | | | | | |
| Week 25 | 0.89 | 0.78 | – | 1.01 | > .05 |  | 0.86 | 0.71 | – | 1.05 | > .05 |  | 0.93 | 0.81 | – | 1.07 | > .05 |  | 1.01 | 0.88 | – | 1.16 | > .05 |  |
|  | | | | | | | | | | | | | | | | | | | | | | | | |
| Week 33 | 0.92 | 0.81 | – | 1.04 | > .05 |  | 0.85 | 0.70 | – | 1.02 | > .05 |  | 0.95 | 0.83 | – | 1.09 | > .05 |  | 1.04 | 0.90 | – | 1.20 | > .05 |  |
|  | | | | | | | | | | | | | | | | | | | | | | | | |
| Week 45 | 0.92 | 0.80 | – | 1.04 | > .05 |  | 0.84 | 0.69 | – | 1.02 | > .05 |  | 0.96 | 0.84 | – | 1.10 | > .05 |  | 1.02 | 0.89 | – | 1.17 | > .05 |  |
|  | | | | | | | | | | | | | | | | | | | | | | | | |
| Week 57 | 0.95 | 0.84 | – | 1.08 | > .05 |  | 0.91 | 0.75 | – | 1.10 | > .05 |  | 0.98 | 0.85 | – | 1.13 | > .05 |  | 1.11 | 0.96 | – | 1.27 | > .05 |  |
|  | | | | | | | | | | | | | | | | | | | | | | | | |

Note: The RR’s are the comparisons of pinaverium and placebo in patients who suffered moderate or severe IBS symptoms (symptoms ≥ 3 in 11 point scales, stool Bristol scale ≥ 5). The *P* values were the results of Chi-square tests.

Table S8. Average Inter-item Correlation (r_∑_) analysis of the primary and secondary endpoints during / post- treatment (ITT)

| **Treatment** | | | | | | | | | |
| --- | --- | --- | --- | --- | --- | --- | --- | --- | --- |
| Day 1 | Pain |  |  | Pain freq |  |  | Stool consist | |  |
|  | r | *P* value |  | r | *P* value |  | r | *P* value |
| Pain frequency | **0.438** | < 0.01 |  |  |  |  |  |  |
| Stool consistency | 0.291 | < 0.01 |  | 0.285 | < 0.01 |  |  |  |
| Stool frequency | 0.176 | < 0.1 |  | 0.024 | > 0.1 |  | **0.212** | < 0.02 |
| Average Inter-item Correlation = | | | | | | | 0.238 | < 0.01 |
| Day 2 | Pain |  |  | Pain freq |  |  | Stool consist |  |
|  | r | *P* value |  | r | *P* value |  | r | *P* value |
| Pain frequency | **0.551** | < 0.01 |  |  |  |  |  |  |
| Stool consistency | 0.211 | < 0.02 |  | 0.275 | < 0.01 |  |  |  |
| Stool frequency | 0.134 | > 0.1 |  | 0.131 | > 0.1 |  | **0.286** | < 0.01 |
| Average Inter-item Correlation = | | | | | | | 0.265 | < 0.01 |
| Day 3 | Pain |  |  | Pain freq |  |  | Stool consist |  |
|  | r | *P* value |  | r | *P* value |  | r | *P* value |
| Pain frequency | **0.566** | < 0.01 |  |  |  |  |  |  |
| Stool consistency | 0.318 | < 0.01 |  | 0.191 | < 0.05 |  |  |  |
| Stool frequency | 0.223 | < 0.02 |  | 0.189 | < 0.05 |  | **0.152** | < 0.1 |
| Average Inter-item Correlation = | | | | | | | 0.273 | < 0.01 |
| Week 1 | Pain |  |  | Pain freq |  |  | Stool consist |  |
|  | r | *P* value |  | r | *P* value |  | r | *P* value |
| Pain frequency | **0.766** | < 0.01 |  |  |  |  |  |  |
| Stool consistency | 0.488 | < 0.01 |  | 0.482 | < 0.01 |  |  |  |
| Stool frequency | 0.353 | < 0.01 |  | 0.368 | < 0.01 |  | **0.468** | < 0.01 |
| Average Inter-item Correlation = | | | | | | | 0.486 | < 0.01 |
| Week 4 | Pain |  |  | Pain freq |  |  | Stool consist |  |
|  | r | *P* value |  | r | *P* value |  | r | *P* value |
| Pain frequency | **0.914** | < 0.01 |  |  |  |  |  |  |
| Stool consistency | 0.680 | < 0.01 |  | 0.610 | < 0.01 |  |  |  |
| Stool frequency | 0.542 | < 0.01 |  | 0.520 | < 0.01 |  | **0.597** | < 0.01 |
| Average Inter-item Correlation = | | | | | | | 0.644 | < 0.01 |
| **Post-treatment** | | | | | | | | | |
| Day 1 | Pain |  |  | Pain freq |  |  | Stool consist |  |  |
|  | r | *P* value |  | r | *P* value |  | r | *P* value |
| Pain frequency | **0.854** | < 0.01 |  |  |  |  |  |  |
| Stool consistency | 0.661 | < 0.01 |  | 0.592 | < 0.01 |  |  |  |
| Stool frequency | 0.552 | < 0.01 |  | 0.446 | < 0.01 |  | **0.595** | < 0.01 |
| Average Inter-item Correlation = | | | | | | | 0.617 | < 0.01 |
| Day 2 | Pain |  |  | Pain freq |  |  | Stool consist |  |
|  | r | *P* value |  | r | *P* value |  | r | *P* value |
| Pain frequency | **0.813** | < 0.01 |  |  |  |  |  |  |
| Stool consistency | 0.729 | < 0.01 |  | 0.612 | < 0.01 |  |  |  |
| Stool frequency | 0.625 | < 0.01 |  | 0.527 | < 0.01 |  | **0.710** | < 0.01 |
| Average Inter-item Correlation = | | | | | | | 0.669 | < 0.01 |
| Day 3 | Pain |  |  | Pain freq |  |  | Stool consist |  |
|  | r | *P* value |  | r | *P* value |  | r | *P* value |
| Pain frequency | **0.807** | < 0.01 |  |  |  |  |  |  |
| Stool consistency | 0.667 | < 0.01 |  | 0.620 | < 0.01 |  |  |  |
| Stool frequency | 0.582 | < 0.01 |  | 0.529 | < 0.01 |  | **0.678** | < 0.01 |
| Average Inter-item Correlation = | | | | | | | 0.647 | < 0.01 |
| Week 4 | Pain |  |  | Pain freq |  |  | Stool consist |  |
|  | r | *P* value |  | r | *P* value |  | r | *P* value |
| Pain frequency | **0.796** | < 0.01 |  |  |  |  |  |  |
| Stool consistency | 0.645 | < 0.01 |  | 0.541 | < 0.01 |  |  |  |
| Stool frequency | 0.594 | < 0.01 |  | 0.444 | < 0.01 |  | **0.651** | < 0.01 |
| Average Inter-item Correlation = | | | | | | | 0.612 | < 0.01 |
| Week 9 | Pain |  |  | Pain freq |  |  | Stool consist |  |
|  | r | *P* value |  | r | *P* value |  | r | *P* value |
| Pain frequency | **0.760** | < 0.01 |  |  |  |  |  |  |
| Stool consistency | 0.641 | < 0.01 |  | 0.557 | < 0.01 |  |  |  |
| Stool frequency | 0.521 | < 0.01 |  | 0.480 | < 0.01 |  | **0.686** | < 0.01 |
| Average Inter-item Correlation = | | | | | | | 0.608 | < 0.01 |
| Week 17 | Pain |  |  | Pain freq |  |  | Stool consist |  |
|  | r | *P* value |  | r | *P* value |  | r | *P* value |
| Pain frequency | **0.753** | < 0.01 |  |  |  |  |  |  |
| Stool consistency | 0.643 | < 0.01 |  | 0.551 | < 0.01 |  |  |  |
| Stool frequency | 0.553 | < 0.01 |  | 0.552 | < 0.01 |  | **0.661** | < 0.01 |
| Average Inter-item Correlation = | | | | | | | 0.619 | < 0.01 |
| Week 25 | Pain |  |  | Pain freq |  |  | Stool consist |  |
|  | r | *P* value |  | r | *P* value |  | r | *P* value |
| Pain frequency | **0.723** | < 0.01 |  |  |  |  |  |  |
| Stool consistency | 0.659 | < 0.01 |  | 0.514 | < 0.01 |  |  |  |
| Stool frequency | 0.537 | < 0.01 |  | 0.446 | < 0.01 |  | **0.611** | < 0.01 |
| Average Inter-item Correlation = | | | | | | | 0.582 | < 0.01 |
| Week 33 | Pain |  |  | Pain freq |  |  | Stool consist |  |
|  | r | *P* value |  | r | *P* value |  | r | *P* value |
| Pain frequency | **0.735** | < 0.01 |  |  |  |  |  |  |
| Stool consistency | 0.596 | < 0.01 |  | 0.452 | < 0.01 |  |  |  |
| Stool frequency | 0.515 | < 0.01 |  | 0.446 | < 0.01 |  | **0.637** | < 0.01 |
| Average Inter-item Correlation = | | | | | | | 0.563 | < 0.01 |
| Week 45 | Pain |  |  | Pain freq |  |  | Stool consist |  |
|  | r | *P* value |  | r | *P* value |  | r | *P* value |
| Pain frequency | **0.704** | < 0.01 |  |  |  |  |  |  |
| Stool consistency | 0.592 | < 0.01 |  | 0.495 | < 0.01 |  |  |  |
| Stool frequency | 0.466 | < 0.01 |  | 0.419 | < 0.01 |  | **0.634** | < 0.01 |
| Average Inter-item Correlation = | | | | | | | 0.552 | < 0.01 |
| Week 57 | Pain |  |  | Pain freq |  |  | Stool consist |  |
|  | r | *P* value |  | r | *P* value |  | r | *P* value |
| Pain frequency | **0.716** | < 0.01 |  |  |  |  |  |  |
| Stool consistency | 0.561 | < 0.01 |  | 0.511 | < 0.01 |  |  |  |
| Stool frequency | 0.494 | < 0.01 |  | 0.451 | < 0.01 |  | **0.633** | < 0.01 |
| Average Inter-item Correlation = | | | | | | | 0.561 | < 0.01 |

Table S9. The number (percentage) of patients with IBS symptoms (ITT)

|  | Pinaverium # (%) |  | Placebo # (%) |  | Chi square *P* value |  | Pinaverium & placebo  # (%) |
| --- | --- | --- | --- | --- | --- | --- | --- |
| **Treatment** | | | | | | | |
| Baseline | 132 (100.0%) |  | 132 (100.0%) |  |  |  |  |
| Day 1 | 68 (51.5%) |  | 111 (84.1%) |  | < 0.001 |  |  |
| Day 2 | 65 (49.2%) |  | 107 (81.1%) |  | < 0.001 |  |  |
| Day 3 | 58 (43.9%) |  | 103 (78.0%) |  | < 0.001 |  |  |
| Week 1 | 55 (41.7%) |  | 88 (67.4%) |  | < 0.001 |  |  |
| Week 4 | 49 (37.1%) |  | 87 (65.9%) |  | < 0.001 |  |  |
| **Post-treatment** | | | | | | | |
| Day 1 | 47 (35.6%) |  | 90 (68.2%) |  | < 0.001 |  |  |
| Day 2 | 47 (35.6%) |  | 92 (69.7%) |  | < 0.001 |  |  |
| Day 3 | 50 (37.9%) |  | 88 (66.7%) |  | < 0.001 |  |  |
| Week 4 | 58 (43.9%) |  | 77 (58.3%) |  | < 0.05 |  |  |
| Week 9 | 54 (40.9%) |  | 75 (56.8%) |  | < 0.01 |  |  |
| Week 17 | 61 (46.2%) |  | 75 (56.8%) |  | > 0.05 |  | 136 (51.5%) |
| Week 25 | 63 (47.7%) |  | 78 (59.1%) |  | > 0.05 |  | 141 (53.4%) |
| Week 33 | 68 (51.5%) |  | 81 (61.4%) |  | > 0.05 |  | 149 (56.4%) |
| Week 45 | 66 (50.0%) |  | 79 (59.8%) |  | > 0.05 |  | 145 (54.9%) |
| Week 57 | 68 (51.5%) |  | 77 (58.3%) |  | > 0.05 |  | 145 (54.9%) |

Notes: IBS symptoms were defined as pain scale ≥ 3 and Bristol stool scale ≥ 5. N = 132

Table S10. Average Inter-item Correlation (r∑) analysis between global overall symptom scales and symptomatic endpoints during / post- treatment (ITT)

|  | Pain | |  | Stool consistency | |  | Pain frequency | |  | Stool frequency | |  | Average (r∑) | |
| --- | --- | --- | --- | --- | --- | --- | --- | --- | --- | --- | --- | --- | --- | --- |
|  | r | *P* value |  | r | *P* value |  | r | *P* value |  | r | *P* value |  | r | *P* value |
|  | | | | | | | | | | | | | | |
| Day 1 | 0.301 | < 0.01 |  | 0.392 | < 0.01 |  | 0.201 | < 0.05 |  | 0.179 | > 0.1 |  | 0.268 | < 0.02 |
| Day 2 | 0.367 | < 0.01 |  | 0.342 | < 0.01 |  | 0.310 | < 0.01 |  | 0.226 | < 0.01 |  | 0.311 | < 0.01 |
| Day 3 | 0.643 | < 0.01 |  | 0.425 | < 0.01 |  | 0.400 | < 0.01 |  | 0.259 | < 0.01 |  | 0.432 | < 0.01 |
| Week 1 | 0.646 | < 0.01 |  | 0.608 | < 0.01 |  | 0.596 | < 0.01 |  | 0.389 | < 0.01 |  | 0.560 | < 0.001 |
| Week 4 | 0.819 | < 0.01 |  | 0.664 | < 0.01 |  | 0.815 | < 0.01 |  | 0.597 | < 0.01 |  | 0.724 | < 0.01 |
|  | | | | | | | | | | | | | | |
| Day 1 | 0.787 | < 0.01 |  | 0.573 | < 0.01 |  | 0.696 | < 0.01 |  | 0.413 | < 0.01 |  | 0.618 | < 0.01 |
| Day 2 | 0.800 | < 0.01 |  | 0.639 | < 0.01 |  | 0.751 | < 0.01 |  | 0.513 | < 0.01 |  | 0.676 | < 0.01 |
| Day 3 | 0.818 | < 0.01 |  | 0.723 | < 0.01 |  | 0.744 | < 0.01 |  | 0.569 | < 0.01 |  | 0.714 | < 0.01 |
| Week 4 | 0.858 | < 0.01 |  | 0.690 | < 0.01 |  | 0.766 | < 0.01 |  | 0.619 | < 0.01 |  | 0.733 | < 0.01 |
| Week 9 | 0.803 | < 0.01 |  | 0.702 | < 0.01 |  | 0.695 | < 0.01 |  | 0.543 | < 0.01 |  | 0.686 | < 0.01 |
| Week 17 | 0.824 | < 0.01 |  | 0.696 | < 0.01 |  | 0.700 | < 0.01 |  | 0.616 | < 0.01 |  | 0.709 | < 0.01 |
| Week 25 | 0.750 | < 0.01 |  | 0.667 | < 0.01 |  | 0.582 | < 0.01 |  | 0.497 | < 0.01 |  | 0.624 | < 0.01 |
| Week 33 | 0.713 | < 0.01 |  | 0.641 | < 0.01 |  | 0.562 | < 0.01 |  | 0.587 | < 0.01 |  | 0.626 | < 0.01 |
| Week 45 | 0.768 | < 0.01 |  | 0.635 | < 0.01 |  | 0.607 | < 0.01 |  | 0.546 | < 0.01 |  | 0.639 | < 0.01 |
| Week 57 | 0.733 | < 0.01 |  | 0.647 | < 0.01 |  | 0.622 | < 0.01 |  | 0.627 | < 0.01 |  | 0.657 | < 0.01 |

Table S11. Patients with at least one treatment-emergent adverse effects (ITT)

|  | Patient number (%) | | | | |
| --- | --- | --- | --- | --- | --- |
|  | Pinaverium | |  | Placebo | |
| Stomach fullness | 3 | 2.27% |  | 2 | 1.52% |
| Headache | 2 | 1.52% |  | 2 | 1.52% |
| Nausea / vomiting | 3 | 2.27% |  | 1 | 0.76% |
| Dizziness | 2 | 1.52% |  | 2 | 1.52% |
| Drowsiness | 2 | 1.52% |  | 0 | 0.00% |
| Heartburn, | 1 | 0.76% |  | 2 | 1.52% |
| Dry mouth | 3 | 2.27% |  | 0 | 0.00% |
| Constipation | 4 | 3.03% |  | 0 | 0.00% |
| Others | 3 | 2.27% |  | 11 | 8.33% |


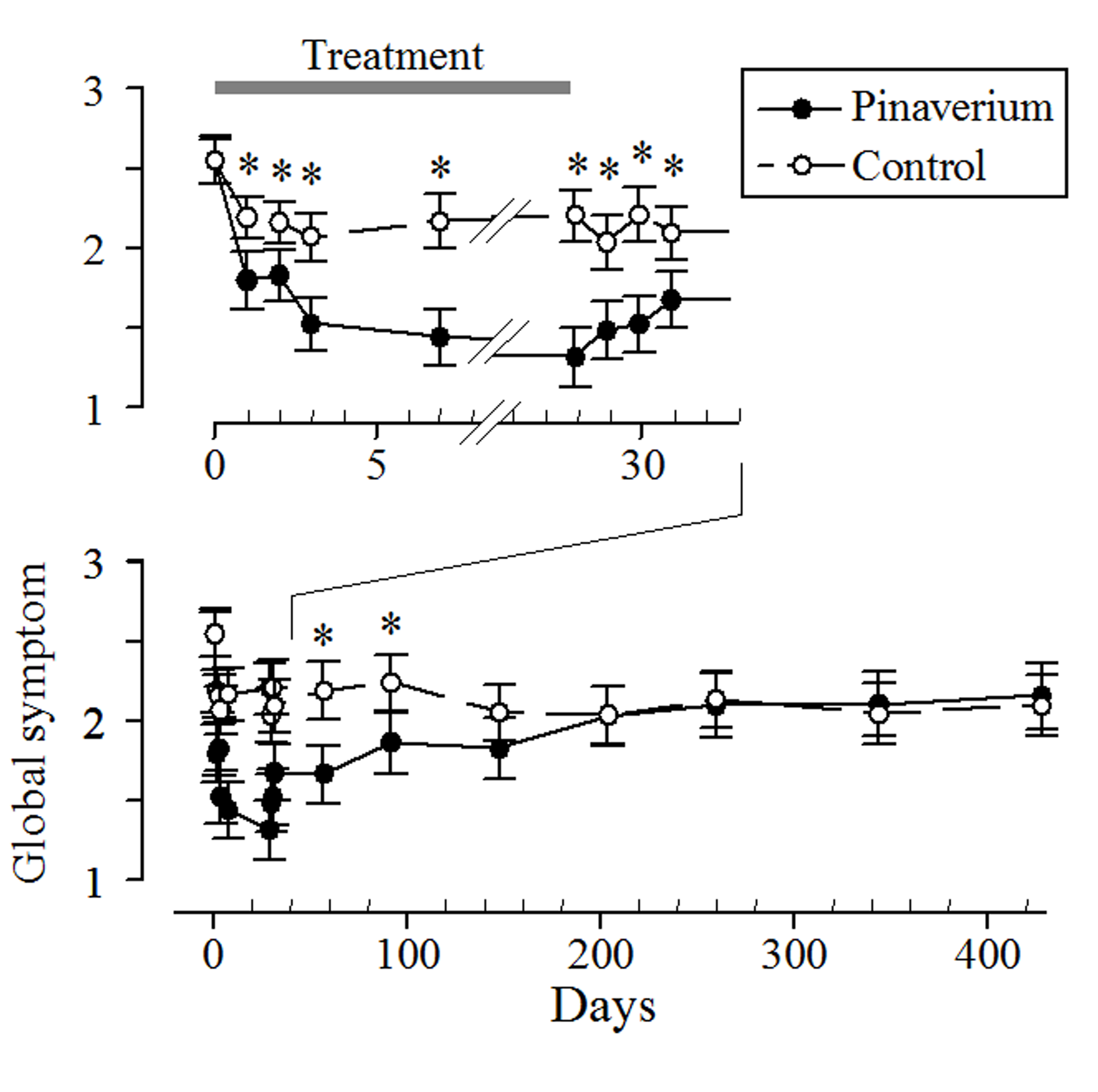


Figure S1. The time course of the global overall symptom during the treatment and post-treatment (intention-to-treat population; n = 132 for each group). Error bars indicate 95% confidence intervals. The t test comparing pinaverium with placebo is indicated by * (P < 0.05).

1. ITT = intention-to-treat population; PP = per-protocol population. [↑](#footnote-ref-2)
2. **References**

   ? Altman DG. Analysis of Survival times. In: Practical statistics for Medical research. London (UK): Chapman and Hall. 1992;pp. 365-93. [↑](#endnote-ref-2)
3. Dudley WN, Wickham R, and Coombs N. An Introduction to Survival Statistics: Kaplan-Meier Analysis. J Adv Pract Oncol. 2016;7:91-100. [↑](#endnote-ref-3)
4. Rich JT, Neely JG, Paniello RC, Voelker CC, Nussenbaum B, Wang EW. A practical guide to understanding Kaplan-Meier curves.Otolaryngol Head Neck Surg. 2010;143:331-6. [↑](#endnote-ref-4)
5. Goel M, Khanna P, Kishore J. Understanding survival analysis: Kaplan-Meier estimate. Int J Ayurveda Res. 2010;1:274-8. [↑](#endnote-ref-5)
6. Staudacher H, Lomer M, Anderson J, Barrett J, Muir J, Irving P, Whelan K. Fermentable carbohydrate restriction reduces luminal bifidobacteria and gastrointestinal symptoms in patients with irritable bowel syndrome. J Nutr. 2012;142:1510-8. [↑](#endnote-ref-6)
7. Pineton G, Neut C, Chau A, Cazaubiel M, Pelerin F, Justen P, Desreumaux P. Saccharomyces in IBScerevisiae versus placebo in the irritable bowel syndrome. Dig Liver Dis. 2015;47:119–24. [↑](#endnote-ref-7)
8. Nobaek S, Johansson M, Molin G, Ahrné S, Jeppsson B. Alteration of intestinal microflora is associated with reduction in abdominal bloating and pain in patients with irritable bowel syndrome. Am J Gastroenterol. 2000;95:1231–8. [↑](#endnote-ref-8)
9. Begtrup L, de Muckadell O, Kjeldsen J, Christensen D, Jarbøl E. Long-term treatment with probiotics in primary care patients with irritable bowel syndrome - a randomized, double-blind, placebo controlled trial. Scand J Gastroenterol. 2013;48:1127–35. [↑](#endnote-ref-9)
10. Lembo A, Pimentel M, Rao S, Schoenfeld P, Cash B, Weinstock L, Paterson C, Bortey E, Forbes W. Repeat treatment with rifaximin is safe and effective in patients with diarrhea-predominant irritable bowel syndrome. Gastroenterology. 2016;151:1113–21. [↑](#endnote-ref-10)
11. Pimentel M, Park S, Mirocha J, Kane S, Kong Y. The effect of a nonabsorbed oral antibiotic (rifaximin) on the symptoms of the irritable bowel syndrome: a randomized trial. Ann Intern Med. 2006;145:557–63. [↑](#endnote-ref-11)
12. Pimentel M, Lembo A, Chey W, Zakko S, Ringel Y, Yu J, Mareya S, Shaw A, Bortey E, Forbes W; TARGET Study Group. Rifaximin therapy for patients with irritable bowel syndrome without constipation. N Engl J Med. 2011;364:22–32. [↑](#endnote-ref-12)
13. Clavé P, Acalovschi M, Triantafillidis JK, Uspensky YP, Kalayci C, Shee V, Tack J; OBIS Study Investigators. randomized clinical trial: otilonium bromide improves frequency of abdominal pain, severity of distention and time to relapse in patients with irritable bowel syndrome. Aliment Pharmacol Ther. 2011;34:432-42. [↑](#endnote-ref-13)
14. Moaffa-Jahromi M, Lankarani KB, Pasalar M, Afsharypuor S, Tamaddon A. Efficacy and safety of enteric coated capsules of anise oil to treat irritable bowel syndrome. J Ethnopharmacol. 2016;194:937–46. [↑](#endnote-ref-14)
15. Cappello G, Spezzaferro M, Grossi L, Manzoli L, Marzio L. Peppermint oil (Mintoil) in the treatment of irritable bowel syndrome: a prospective double blind placebo-controlled randomized trial. Dig Liver Dis. 2007;39:530–6. [↑](#endnote-ref-15)
16. Vahedi H, Merat S, Rashidioon A, et al. The effect of fluoxetine in patients with pain and constipation-predominant irritable bowel syndrome: a double-blind randomized-controlled study. Aliment Pharmacol Ther. 2005;22:381–5. [↑](#endnote-ref-16)
17. Payne A & Blanchard E. A controlled comparison of cognitive therapy and self-help support groups in the treatment of irritable bowel syndrome. J Consult Clin Psychol. 1995;63:779–86. [↑](#endnote-ref-17)
18. Tkachuk G, Graff L, Martin G, Bernstein C. Randomized controlled trial of cognitive-behavioral group therapy for irritable bowel syndrome in a medical setting. J Clin Psychol Med Settings. 2003;10:57–69. [↑](#endnote-ref-18)
19. Keefer L, Blanchard E. The effects of relaxation response meditation on the symptoms of irritable bowel syndrome: results of a controlled treatment study. Behav Res Ther. 2001;39:801–11. [↑](#endnote-ref-19)
20. Lynch P and Zamble E. A controlled behavioral treatment study of irritable bowel syndrome. Behav Ther. 1989;20:509–23. [↑](#endnote-ref-20)
21. Lindfors P, Unge P, Arvidsson P, Nyhlin H, Björnsson E, Abrahamsson H, Simrén M. Effects of gut-directed hypnotherapy on IBS in different clinical settings - Results from two randomized, controlled trials. Am J Gastroenterol. 2012;107:276–85. [↑](#endnote-ref-21)
22. Heitkemper M, Jarrett M, Levy R, Cain K, Burr R, Feld A, Barney P, Weisman P. Self-management for women with irritable bowel syndrome. Clin Gastroenterol Hepatol. 2004;2:585–96. [↑](#endnote-ref-22)
23. Moss-Morris R, McAlpine L, Didsbury L, Spence M. A randomized controlled trial of a cognitive behavioral therapy-based self-management intervention for irritable bowel syndrome in primary care. Psychol Med. 2010;40:85–94. [↑](#endnote-ref-23)
24. Hunt M, mohier S, Milonova M. Brief cognitive-behavioral internet therapy for irritable bowel syndrome. Behav Res Ther. 2009;47:797–802. [↑](#endnote-ref-24)
25. Ljotsson B, Falk L, Wibron Vesterlund A, Hedman E, Lindfors P, Rück C, Hursti T, Andréewitch S, Jansson L, Lindefors N, Andersson G. Internet-delivered exposure and mindfulness based therapy for irritable bowel syndrome - A randomized controlled trial. Behav Res Ther. 2010;48:531–9. [↑](#endnote-ref-25)
26. C reed F, Fernandes L, Guthrie E, Palmer S, Ratcliffe J, Read N, Rigby C, Thompson D, Tomenson B; North of England IBS Research Group. The cost-effectiveness of psychotherapy and paroxetine for severe irritable bowel syndrome. Gastroenterology. 2003;124:303–17. [↑](#endnote-ref-26)
27. Zernicke K, Campbell T, Blustein P, Fung T, Johnson J, Bacon S, Carlson L. Mindfulness-based stress reduction for the treatment of irritable bowel syndrome symptoms: A randomized wait-list controlled trial. Int J Behav Med. 2013;20:385–96. [↑](#endnote-ref-27)
28. Gaylord S, Palsson O, Garland E, Faurot K, Coble R, Mann J, Frey W, Leniek K, Whitehead W. Mindfulness training reduces the severity of irritable bowel syndrome in women: results of a randomized controlled trial. Am J Gastroenterol. 2011;106:1678–88. [↑](#endnote-ref-28)
29. Lackner J, Jaccard J, Krasner S, Katz L, Gudleski G, Holroyd K. Self-administered cognitive behavior therapy for moderate to severe irritable bowel syndrome: clinical efficacy, tolerability, feasibility. Clin Gastroenterol Hepatol. 2008;6:899–906. [↑](#endnote-ref-29)
30. Jarrett M, Cain K, Burr R, Hertig V, Rosen S, Heitkemper M. Comprehensive self-management for irritable bowel syndrome: randomized trial of in-person vs. combined in-person and telephone sessions. Am J Gastroenterol. 2009;104:3004–14. [↑](#endnote-ref-30)
31. Camilleri M, Northcutt A, Kong S, Dukes G, McSorley D, Mangel A. Efficacy and safety of alosetron in women with irritable bowel syndrome: a randomized, placebo-controlled trial. Lancet. 2000;355:1035–40. [↑](#endnote-ref-31)
32. Camilleri M, Chey W, Mayer E, Northcutt A, Heath A, Dukes G, McSorley D, Mangel A. A randomized controlled clinical trial of the serotonin type 3 receptor antagonist alosetron in women with diarrhea-predominant irritable bowel syndrome. Arch Intern Med. 2001;161:1733–40. [↑](#endnote-ref-32)
33. Lembo T, Wright R, Bagby B, Decker C, Gordon S, Jhingran P, Carter E; Lotronex Investigator Team. Alosetron controls bowel urgency and provides global symptom improvement in women with diarrhea-predominant irritable bowel syndrome. Am J Gastroenterol. 2001;96:2662–70. [↑](#endnote-ref-33)
34. Chey W, Chey W, Heath A, Dukes G, Carter E, Northcutt A, Ameen V. Long-term safety and efficacy of alosetron in women with severe diarrhea-predominant irritable bowel syndrome. Am J Gastroenterol. 2004;99:2195-203. [↑](#endnote-ref-34)
35. Chang L, Ameen V, Dukes G, McSorley D, Carter E, Mayer E. A dose-ranging, phase II study of the efficacy and safety of alosetron in men with diarrhea-predominant IBS. Am J Gastroenterol. 2005;100:115–23. [↑](#endnote-ref-35)
36. Barbara G, Cremon C, Annese V, Basilisco G, Bazzoli F, Bellini M, Benedetti A, Benini L, Bossa F, Buldrini P, Cicala M, Cuomo R, Germanà B, Molteni P, Neri M, Rodi M, Saggioro A, Scribano ML, Vecchi M18, Zoli G, Corinaldesi R, Stanghellini V. randomized controlled trial of mesalazine in IBS. Gut. 2016;65:82–90. [↑](#endnote-ref-36)
37. Henrich J, Gjelsvik B, Surawy C, Evans E, Martin M. A randomized clinical trial of mindfulness-based cognitive therapy for women with irritable bowel syndrome-Effects and mechanisms. J Consult Clin Psychol. 2020;88:295-310. [↑](#endnote-ref-37)
38. Shin S, Cha B, Kim W, Park J, Kim J, Choi C. The Effect of Phloroglucinol in Patients With Diarrhea-predominant Irritable Bowel Syndrome: A Randomized, Double-blind, Placebo-controlled Trial. J Neurogastroenterol Motil. 2020;26:117-27. [↑](#endnote-ref-38)
39. Everitt H, Landau S, O'Reilly G, Sibelli A, Hughes S, Windgassen S, Holland R, Little P, McCrone P, Bishop F, Goldsmith K, Coleman N, Logan R, Chalder T, Moss-Morris R; ACTIB trial group. Assessing telephone-delivered cognitive-behavioral therapy (CBT) and web-delivered CBT versus treatment as usual in irritable bowel syndrome (ACTIB): a multicentre randomized trial. Gut. 2019;68:1613-23. [↑](#endnote-ref-39)
